# Systematics of *Disakisperma* (Poaceae, Chloridoideae, Chlorideae)

**DOI:** 10.3897/phytokeys.26.5649

**Published:** 2013-09-27

**Authors:** Neil Snow, Paul M. Peterson, Konstantin Romaschenko

**Affiliations:** 1 Department of Biology, Pittsburg State University, Pittsburg, KS 66762 USA; 2 Department of Botany MRC-166, National Museum of Natural History, Smithsonian Institution, Washington, DC 20013-7012 USA; 3 M.G. Kholodny Institute of Botany, National Academy of Sciences, 01601 Kiev, Ukraine

**Keywords:** Conservation, *Coelachyrum*, *Cypholepis*, *Disakisperma*, grasses, ITS, leaf anatomy, lectotypification, *Leptochloa*, phylogeny, plastid DNA sequences, stem anatomy, systematics, taxonomy, Thysanoptera

## Abstract

*Disakisperma* Steud. is a genus of four predominantly perennial C_4_ (NAD-ME) species in the Americas, Africa, and Asia. Its species previously were treated in *Eleusine*, *Eragrostis*, *Coelachyrum*, *Cypholepis*, *Leptochloa*, or *Diplachne* by nearly all authors.It includes the widespread North and South American amphitropical disjunct *Disakisperma dubium* (type of the genus), *Disakisperma eleusine* from southern Africa, *Disakisperma obtusiflorum* from central and northern Africa to southern Asia, and *Disakisperma yemenicum*, **comb. nov.** from eastern and southern Africa to Yemen. This paper provides a key to the species, geographic distributions, descriptions, including comments on the anatomy of leaves, stems, lemmatal micromorphology, a phylogram based on five molecular markers, and discussions of chromosome numbers. The species are rarely, if at all, known outside of their native ranges and are unlikely to become aggressively invasive. All species are considered Least Concern following IUCN guidelines. Lectotypes are designated for *Diplachne dubia* var. *pringleana* Kuntze, *Disakisperma mexicana* Steud., *Eragrostis yemenica* Schweinf., and *Leptochloa appletonii* Stapf.

## Abstract (es)

*Disakisperma* Steud. es un género de quatro especies perennes C_4_ (NAD-ME), las cuales que son de las Américas y África y Asia. Sus especies previamente fueron tratados en *Eleusine*, *Eragrostis*, *Coelachyrum*, *Cypholepis*, *Leptochloa*, o *Diplachne* por casi todos los autores. Incluye la generalizada del Norte y América del Sur amfitropical disjunta *Disakisperma dubium* (tipo del género), *Disakisperma eleusine* desde el sur de África, *Disakisperma obtusiflorum* del centro y el norte de África hasta el sur de Asia, y *Disakisperma yemenicum*, **comb. nov.** del este y sur África a Yemen. Este documento proporciona una clave para las especies y las descripciones completas, incluidos los comentarios sobre la anatomía de las hojas, tallos, micromorfología lemmatal, una filograma basado en cinco marcadores moleculares y las discusiones de los números de cromosomas. Las especies son raramente, si acaso, conocida fuera de sus áreas de distribución natural y es poco probable para convertirse en invasora agresiva. Todos las epecies se consideran la designación de Least Concern sigue IUCN. Se designa lectotipos para *Diplachne dubia* var. *pringleana* Kuntze, *Disakisperma mexicana* Steud., *Eragrostis yemenica* Schweinf. y *Leptochloa appletonii* Stapf.

## Introduction

Recent molecular studies by Peterson et al. (2010, 2012) determined *Leptochloa* P. Beauv. s.l. (Snow 1997) to be polyphyletic, with its sampled species partitioned into five strongly supported clades. Peterson et al. (2012) analyzed 22 of the 32 species of *Leptochloa* and a wide representation of taxa from related genera in subfamily Chloridoideae, tribe Chlorideae. *Disakisperma* Steud. was one of the proposed segregate genera (Peterson et al. 2012), but that generic name has not been used widely since its description by Steudel (1854). Nearly all authors have placed *Disakisperma* in *Leptochloa* or *Diplachne* (see summaries in Valls 1978; McNeill 1979; Snow 1997).

The clade in Peterson et al. (2012) that we recognize as *Disakisperma* included *Leptochloa dubia* (Kunth) Nees, *Leptochloa eleusine* (Nees) Cope & N. Snow, and *Leptochloa obtusiflora* Hochst. These were the first molecular results to suggest a close relationship among these species, although based on overall morphology Steudel (1841: 30) placed *Leptochloa obtusiflora* Trin. ex Steud. (nom. inval.) as a synonym of *Leptochloa dubia*, suggesting their affinity. In a paper documenting C_4_ origins in the grasses, Aliscioni et al. (2012) presented a summary tree for Chloridoideae derived from *rbcL, ndhF*, and *trnK/matK* sequences, which united *Leptochloa dubia* and *Coelachyrum yemenicum* (Schweinf.) S.M. Phillips in a clade with moderate bootstrap support. For this study we included two samples of *Coelachyrum yemenicum* to test its relationship with other members of *Disakisperma*.

The genus *Disakisperma* was first described by Steudel (1854) based on *Disakisperma mexicana* Steud.; the latter name probably first placed in synomymy of *Leptochloa dubia* by Chase and Niles (1962). The biology and taxonomy of *Disakisperma dubium* (Kunth) P. M. Peterson & N. Snow have been studied in some detail by Valls (1978), albeit with different interpretations of how many infraspecfic taxa to recognize (Nicora 1995; Snow 1997, 2012), whereas knowledge of the African species is more limited (Phillips 1974a; Gibbs Russell et al. 1991; Snow 1997). Historically, the classification of the four species included in our study has been disparate. *Disakisperma dubium* was first describedas *Chloris dubia* by Kunth (1816); *Disakisperma eleusine* (Nees) P.M. Peterson & N. Snow was first recognized as *Diplachne eleusine* by Nees von Esenbeck (1841); the basionym of *Disakisperma obtusiflorum* (Hochst.) P.M. Peterson & N. Snow is *Leptochloa obtusiflora*, first described by Hochstetter (1855); and *Coelachryum yemenicum* was first described by Schweinfurth (1894) as an *Eragrostis*, placed in the monotypic *Cypholepis* by Chiovenda (1908), who transferred it to *Eleusine* (Chiovenda 1912), and finally Phillips (1982), who transferred it to *Coelachyrum*.

The purpose of this paper is to present a systematic account of *Disakisperma* as part of a series of papers separating *Leptochloa* s.l. (Snow 1997) into monophyletic genera (see also Snow and Peterson 2012). In addition, we include a phylogram derived from analysis of combined plastid and ITS sequences that suggests evolutionary relationships among the four species of *Disakisperma*.

## Materials and methods

We viewed over 1500 herbarium specimens from nearly 60 herbaria for this revision (see Acknowledgements). However, we estimate that less than half of the existing specimens of *Disakisperma dubium* have been cited, particularly in the southwestern USA and northwestern Mexico, given the large numbers of collections deposited in the larger herbaria (e.g., ARIZ, ASU, MEXU, TAES, TEX) and in many smaller herbaria that we have not visited in North America and southern South America. The first author has collected a dozen specimens of *Disakisperma dubium* in the USA and parts of Mexico and *Disakisperma eleusine* in South Africa, whereas the second author has collected 85 specimens of *Disakisperma dubium* across much of its native range, and *Disakisperma obtusiflorum* (3) and *Coelachryum yemenicum* in Tanzania. Geographic range abbreviations follow Brummitt (2001) and herbarium abbreviations follow Thiers (2013).

Fresh leaf and stem material was preserved for anatomical analysis and studied for (but not summazied) in Snow (1997). Leaf anatomy terminology follows Ellis (1976). Characters of lemmatal micromorphology were studied using scanning electron microscopy (Snow 1996), and caryopsis features were viewed using simple light microscopy (Snow 1998b).

**Figure 1.**
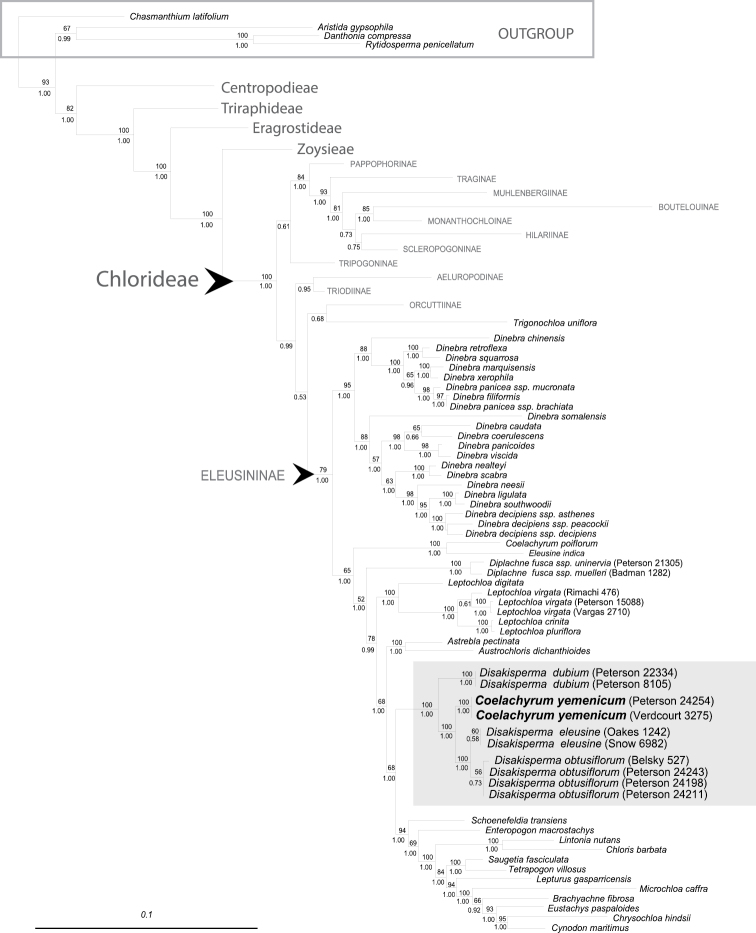
Phylogram of maximum-likelihood tree from analysis of combined plastid (*rpL32-trnL*, *ndhA* intron, *rps16* intron, and *rps16-trnK*) and ITS sequences. Numbers above branches represent bootstrap values; numbers below branches represent posterior probabilities; gray highlighted area is the *Disakisperma* (incl. *Coelachyrum yemenicum*) clade. Scale bar = 10% sequence divergence.

The phylogram (Fig. 1) was generated with existing data from Peterson et al. (2012) but with six new samples added: three of *Disakisperma obtusiflorum*, two of *Coelachyrum yemenicum*, andone of *Disakisperma eleusine*. Voucher information and GenBank numbers for the new samples are given in Table 1. The methods for DNA extraction, amplification, sequencing, and phylogenetic analysis are given in Peterson et al. (2012). We estimated the phylogeny among members of *Disakisperma* based on the analysis of six molecular markers (nuclear ITS 1&2 and plastid *rpL32-trnL*, *ndhA* intron, *rps16* intron, and *rps16-trnK* DNA sequences). To make the phylogram smaller some clades are depicted at a higher level (subtribe or tribe) and the number of species in each is: Aeluropodinae (1), Boutelouinae (1), Centropodieae (1), Eragrostideae (9), Hilariinae (2), Monanthochoinae (3), Muhlenbergiinae (2), Orcuttiinae (2), Pappophorinae (2), Scleropogoninae (3), Traginae (3), Triodiinae (2), Tripogoninae (4), Triraphideae (2), and Zoysieae (5).

**Table 1.** Specimens sampled in the *Disakisperma*−*Coelachryum yememicum* clade, vouchers (MO = Missouri Botanical Garden; US = United States National Herbarium, Smithsonian Institution), country of origin, and GenBank accession numbers for DNA sequences. All accessions marked in **bold** are newly submitted sequences to GenBank.<br/>

**Taxon**	**Voucher**	**Country**	**rpl32-trnL**	**ndhA intron**	**rps16 intron**	**rps16-trnK**	**ITS**
*Disakisperma dubium* (Kunth) P.M. Peterson & N. Snow	Peterson 22334 & Saarela (US)	Mexico	GU359811	GU359442	GU360416	GU360695	GU359145
*Disakisperma dubium* (Kunth) P.M. Peterson & N. Snow	Peterson 8105 & Annable (US)	Mexico	JQ345332	JQ345214	JQ345290	JQ345247	JQ345179
*Disakisperma eleusine* (Nees) P.M. Peterson & N.Snow	Snow 6982 (MO)	South Africa	JQ345333	JQ345215	JQ345291	JQ345248	JQ345180
*Disakisperma eleusine* (Nees) P.M. Peterson & N.Snow	Oakes 1242 (US)	South Africa	**KF574410**		**KF574416**	**KF574422**	**KF574399**
*Disakisperma obtusiflorum* (Hochst.) P.M. Peterson & N. Snow	Peterson 24243, Soreng & Romaschenko (US)	Tanzania	**KF574413**	**KF574407**	**KF574419**	**KF574425**	**KF574402**
*Disakisperma obtusiflorum* (Hochst.) P.M. Peterson & N. Snow	Belsky 527 (MO)	Kenya	JQ345340		JQ345298	JQ345255	JQ345187
*Disakisperma obtusiflorum* (Hochst.) P.M. Peterson & N. Snow	Peterson 24198, Soreng & Romaschenko (US)	Tanzania	**KF574411**	**KF574405**	**KF574417**	**KF574423**	**KF574400**
*Disakisperma obtusiflorum* (Hochst.) P.M. Peterson & N. Snow	Peterson 24211, Soreng & Romaschenko (US)	Tanzania	**KF574412**	**KF574406**	**KF574418**	**KF574424**	**KF574401**
*Disakisperma yemenicum* (Schweinf.) P.M. Peterson & N. Snow	Peterson 24254, Soreng & Romaschenko (US)	Tanzania	**KF574414**	**KF574408**	**KF574420**	**KF574426**	**KF574403**
*Disakisperma yemenicum* (Schweinf.) P.M. Peterson & N. Snow	Verdcourt 3275 (US)	Kenya	**KF574415**	**KF574409**	**KF574421**	**KF574427**	**KF574404**

## Results and discussion

**Phylogeny.** A total of 29 sequences from six species are newly reported in GenBank (Table 1). Total aligned characters for individual regions are noted in Table 2. We combined the plastid−ITS sequences in our analysis since there were acceptable levels of congruence between the majority of the data sets in Peterson et al. (2012).

**Table 2.** Summary of the four plastid and nrDNA ITS regions used in the maximum likelihood and Bayesian searches indicated by Akaike’s Information Criterion (AIC).

**Characteristic**	***rpL32-trnL***	***ndhA intron***	***rps16 intron***	***rps16-trnK***	**Combined plastid data**	***ITS***	**Overall combined dataset**
Total aligned characters	1233	1323	1074	1040	4670	832	5502
Maximum likelihood scores (-lnL)	9567.4667	8778.3245	6732.027	8265.2405		20947.0843	
Number of substitution types	6	6	6	6		6	
Model for among−site rate variation	gamma	gamma	gamma	gamma		gamma	
Substitution rates	0.9361<br/> 1.8439<br/> 0.4825<br/> 1.4468<br/> 1.5696<br/> 1.0000	1.3591<br/> 2.8338<br/> 0.5457<br/> 2.2589<br/> 2.9694<br/> 1.0000	1.0865<br/> 1.4745<br/> 0.3367<br/> 1.3805<br/> 2.2541<br/> 1.0000	1.1338<br/> 2.6853<br/> 0.5231<br/> 1.7173<br/> 2.4576<br/> 1.0000		1.2977<br/> 2.6471<br/> 1.4264<br/> 0.9278<br/> 4.9114<br/> 1.0000	
Character state frequencies	0.3836<br/> 0.1431<br/> 0.1295<br/> 0.3436	0.3767<br/> 0.1249<br/> 0.1430<br/> 0.3552	0.3925<br/> 0.1047<br/> 0.1636<br/> 0.3390	0.3066<br/> 0.1341<br/> 0.1407<br/> 0.4184		0.2495<br/> 0.1914<br/> 0.2496<br/> 0.3093	
Proportion of invariable sites	0.0774	0.2175	0.1551	0.1303		0.2365	
Substitution model	GTR+I+G	TVM+G	TIM3+I+G	TIM3+G		GTR+I+G	
Gamma shape parameter (α)	0.9636	1.2427	1.1594	1.6560		0.9959	

The maximum-likelihood tree from the combined analysis of four plastid regions (*rpL32-trnL*, *ndhA* intron, *rps16* intron, and *rps16-trnK*) and ITS is well resolved, with strong to moderate support for the tribes and most subtribes of the Chloridoideae (Fig. 1). The Chloridoideae is composed of five tribes; followed by, in order of divergence: Centropodieae, Triraphideae, Eragrostideae, Zoysieae, and Chlorideae (Peterson et al. 2010, 2011, 2012). Within the subtribe Eleusininae, species of *Leptochloa* s.l. form four major clades: *Dinebra*, *Diplachne*, *Leptochloa* s.s., and *Disakisperma* (including *Coelachyrum yemenicum*). *Trigonochloa*, a genus with only two species (formerly placed in *Leptochloa*) is sister to the Orcuttiinae; and together these are sister to the Eleusininae (Peterson et al. 2012; see also Snow and Peterson 2012). In the *Disakisperma* clade (incl. *Coelachyrum yemenicum*; bootstrap: BS = 100, posterior probability: PP = 1.00), *Disakisperma eleusine* and *Disakisperma obtusiflorum* are sister (BS = 100, PP = 1.00); *Coelachyrum yemenicum* is sister to these (BS = 100, PP = 1.00); and *Disakisperma dubium* is sister to all three species (BS = 100, PP = 1.00) [Fig. 1].

**Morphology.** *Disakisperma* (including *Coelachyrum yemenicum*) can be recognized by its paniculate inflorescence (=synflorescence) composed of several unilateral racemes that are racemosely to subdigitally inserted along a central axis, spikelets with multiple florets (4−14), mostly 3-nerved lemmas and 1-nerved glumes, and dorsally flattened, mostly elliptical caryopses that are shallow to broadly concave on the hilar surface with a pericarp weakly adnate to endosperm.

**Lemmatal micromorphology.** The four species share a similar but not identical suite of lemma micromorphological traits, including the presence of silica cells, cork cells, long cells and short cells, and bicellular microhairs. The lemmatal hairs of *Disakisperma eleusine*, *Disakisperma obtusiflorum* and *Disakisperma yemenicum* are clavicorniculate (apically club-shaped with a pointed tip), whereas those of *Disakisperma dubium* are acute or rounded apically (Snow 1996). These four species form a strongly supported clade in the phylogram (Fig. 1).

**Caryopsis morphology.** The hilar profile of the caryopsis in *Disakisperma* is elliptic, with the most common length:width ratio being approximately 2:1 (Snow 1998b). However, an elliptic hilar profile occurs in many genera of Chloridoideae (Snow 1998b). Variation in hilar profile shape and length was noted for *Disakisperma dubium* (Valls 1978), which reflects its wide geographical range and considerable variation in vegetative and reproductive morphology (Valls 1978; Snow 1997, 2003, 2012). For example, the hilar profile of *Gould 12183* (K) from Baja California Sur in Mexico was widely obovate (Snow 1998b). In contrast, a narrowly elliptic hilar profile of *Disakisperma dubium* was observed for *Warnock 46783* (NCU) from Pecos County, Texas. Variation in hilar profiles also was observed for *Disakisperma eleusine* (Snow 1998b), which also sometimes had an obovate profile (i.e., broadened towards the apex; e.g., *Drège s.n.* (S) and *Extension Officer 16419* PRE).The hilar profile of *Coelachyrum yemenicum* is elliptic (Snow 1998b). Valls (1978) noted considerable variation in caryopsis morpholgy among the cleistogenous spikelets of *Disakisperma dubium* that occur within the leaf sheath, but Snow (1998b) did not measure variation among cleistogamous spikelets.

In transverse section the caryopsis of *Disakisperma* is usually transversely elliptic (Snow 1998b). This shape accords with many previous observations that the spikelets are strongly flattened dorsally in species now treated in *Diakisperma* (Parodi 1927; McNeill 1979; Lazarides 1980; Phillips 1982; Jacobs 1988; Nowack 1994; Nicora 1995). Snow (1998b) also reported “rounded shallowly obtriangular” for *Disakisperma eleusine*, but in reviewing his original notes for that project, now considers that report erroneous.

In addition to its transversely elliptic shape, all four species typically (but not always) have caryopses that are concave on the hilar surface (Snow 1998b). For example, in *Disakisperma dubium* the broad shallow concavity was noted for *Warnock 46783* from Texas(NCU), *Kral 51801* from Florida (MO), and *Hernández & Mathia N-2066* from San Luis Potosí, Mexico (GH). The depression was not noted specifically for *Mearns 1213* (US) from Kinney County, Texas; *Gould 12183* (K) from Baja California Sur, or *Castillon 43560* (GH) from Tucumán, Argentina, although it probably was shallow in these. Broad to shallow concavities were noted to characterize the caryopses of some species for all specimens observed by Snow (1998b) for *Disakisperma eleusine* and *Disakisperma obtusiflorum* (and, newly noted here for the latter, *Blesky 433* (BH) from Kenya). However, broad to shallow depressions were noted for many specimens of species treated by Snow (1997) in *Leptochloa* s.l., including: *Dinebra aquatica* (Scribn. & Merr.) P.M. Peterson & N. Snow: *Pringle 6664* (US); *Dinebra chinensis* (L.) P.M. Peterson & N. Snow: *Davidse 7471* (MO) from Sri Lanka; *Dinebra decipiens* (R. Br.) P.M. Peterson & N. Snow subsp. *decipiens*: *Lazarides 5634* (US) from Queensland, Australia; *Dinebra decipiens* subsp. *peacockii* (Maiden & Betche) P.M. Peterson & N. Snow: *R. Johnson 713* (CANB) from Queensland, Australia; *Dinebra panicoides* (J. Presl) P.M. Peterson & N. Snow: *Francoer & Williams 47* (ENCB) from Oaxaca, Mexico; *Dinebra viscida* (Scribn.) P.M. Peterson & N. Snow: *Palmer 1789* (GH) from Sinaloa, Mexico; *Leptochloa digitata* (R. Br.) Domin.: *Blake 6320* (CANB) from Queensland, Australia; *Leptochoa longa* Griseb.: *Davidse 2612* (MO) from Trinidad; and *Coelachyrum yemenicum*: *Schweickerdt 2011* (S) origin not recorded.

The pericarp is weakly adnate among species of *Disakisperma* and disassociates relatively quickly in water at room temperature (Snow 1998b). This is a homoplastic trait within Chlorideae given its recurrence in *Diplachne* s.s. (Snow 1997, 1998b; Peterson et al. 2012), *Dinebra panicoides*, and *Dinebra viscida*, the latter two of which form a distinct clade in the combined plastid and ITS phylogram (Fig. 3 in Peterson et al. 2012). *Leptochloa chloridiformis* (Hack. ex Stuck.) Parodi, in the newer and narrower circumscription of *Lepotchloa* (Peterson et al. 2012), was the only species in which the pericarp readily disascociated in water (Snow 1998b). Valls (1978: 104) also reported a freely disassociating pericarp for the rarely collected *Leptochloa longa* Griseb., of which we have not yet obtained molecular data.

The hilum is uniformly punctiform and highly conspicuous among species historically treated in *Leptochloa* s.l. (including Snow 1997). Thus hilar shape is of little if any value in the generic reconfiguring of *Leptochloa*.

To summarize, when used with other characters, species of *Disakisperma* generally can be characterized by the dorsally flattened caryopses that are broadly to shallowly concave on the hilar side, with pericarps that detach readily from the seed wall of the caryopsis in water. However, since some species in closely related chloridoid genera also share these homoplastic traits, features of the caryopsis by themselves are insufficient to diagnose *Disakisperma*. This observation echoes Vavilov (1922) and his “Law of Homologous Series in Variation”. However, the term homologous is a misnomer as presently used by evolutionary biologists, given that such traits are now considered non-homologous (homoplastic).

## Taxonomic treatment

### Disakisperma

Steud., Syn. Pl. Glumac. 1: 287. 1854.

http://species-id.net/wiki/Disakisperma

#### Type species.

*Disakisperma mexicana* Steud. = *Disakisperma dubium* (Kunth) P.M. Peterson & N. Snow.

#### Description.

Plants perennial, rarely annual in a few populations, occasionally stoloniferous. Culms 30–200 cm long, solid, decumbent or clambering to erect; nodes glabrous. Leaf sheaths half as long to slightly longer than internodes, glabrous or ciliate apically along margins; ligule membranous, 0.5–1.5 mm long, ciliate or fimbriate apically; leaf blades cauline, linear. Inflorescence apical and exserted at maturity or cleistogamous in lower leaf sheaths, a panicle composed of several to numerous unilateral racemes, racemosely or subdigitately scattered along a central axis; branches at maturity slightly reflexed to ascending or steeply erect. Spikelets sessile to subsessile, dorsally rounded to flattened, typically overlapping, disarticulation above the glumes; florets 4−13; glumes 2, 1-nerved or occasionally with remnants of two additional nerves near base, mucronate or emucronate; lemmas 3-nerved, rarely with remnants of two additional nerves near base, sometimes cartilaginous towards the base, macrohairs acute, obtuse, or clavicorniculate; paleas often somewhat cartilaginous towards base. Stamens 3. Lodicules 2, flabellate. Caryopses dorsally flattened, broadly concave on the hilar surface; pericarp weakly adnate to endosperm. 2*n* = 40, 60, 80 (Snow 1997).

#### Vernacular name.

In light of its only recent resurrection from generic synonymy (Peterson et al. 2012), no common name exists for *Disakisperma*. We suggest Jacobsgrass to honor the memory of Dr. Surrey W. L. Jacobs (1946−2009), an Australian friend, colleague, and chloridoid specialist (e.g., Jacobs 1988).

#### Key to the species of *Disakisperma*

**Table:** 

1	Panicles 1–3 cm wide; branches mostly erect or steeply ascending, stiff, 2–10.5 cm long	2
–	Panicles 3–25 cm wide; branches ascending but not steeply so, usually reflexed (towards tips) and somewhat flexuous, (−1.5) 3−19 cm long.	3
2	Lemmas membranous throughout, margins not involute near base; adaxial leaf blades surfaces without long hairs; anthers 0.9−1.0 mm long	*Disakisperma eleusine*
–	Lemmas cartilaginous below, margins involute near base; adaxial leaf blade with scattered, delicate, straight hairs near the base, the hairs 3−5 mm long; anthers 0.2−0.3 mm long	*Disakisperma yemenicum*
3	Lemmas 3.5−5.0 mm long with round- to acute-tiped hairs along margins and sometimes the midnerve below; glumes 3.3−6.0 mm long; cleistogamous spikelets hidden in lower leaf sheaths; anthers 1.0−1.6 mm long; native to the Americas	*Disakisperma dubium*
–	Lemmas 2.2−2.8 (−3.0) mm long with clavicorniculate (club-shaped) hairs along the margins and midnerve below; glumes 1.5−2.9 mm long; cleistogamous spikelets absent in the lower leaf sheaths; anthers about 0.7 mm long; native to Africa and Asia	*Disakisperma obtusiflorum*

### Disakisperma dubium

(Kunth) P.M. Peterson & N. Snow, Ann. Bot., 109: 1327. 2012.

http://species-id.net/wiki/Disakisperma_dubium

Figure 2A−P

Chloris dubia Kunth, Nov. Gen. Sp. 1: 169. 1816. *Leptostahys dubia* (Kunth) G. Mey., Prim. Fl. Esseq. 74. 1818. *Leptochloa dubia* (Kunth) Nees, Syll. Pl. Nov. 1: 4. 1824. *Festuca obtusiflora* Willd. ex Spreng., Syst. Veg. 1: 356. 1825. *Diplachne dubia* (Kunth) Scribn. Bull. Torrey Bot. Club 10: 30. 1883. *Rabdochloa dubia* (Nees) Kuntze ex Stuck., Anales Mus. Nac. Buenos Aires 11: 121. 1904. *Sieglingia dubia* (Kunth) Kuntze ex Stuck., Anales Mus. Nac. Buenos Aires 11: 128. 1904.
Schismus patens J. Presl. Reliq. Haenk. 1(4-5): 269. 1830. *Leptochloa patens* (J. Presl) Kunth, Enum. Pl. 1: 271. 1833. *Diplachne patens* (J. Presl). TYPE: Chile, *Hab. in Cordilleris chilensibus*,T. Haenke s.n.(holotype: PR; isotype: US-A78816!).
Disakisperma mexicana Steud., Syn. Pl. Glumac. 1: 287. 1854. (“1855”). TYPE: Mexico, Vallee de Mexico, Aug 1827, J.L. Berlandier 758 (lectotype: P ex herb. Drake barcode P02295597 seen digitally!; isolectotypes: MO-134708 seen digitally!, US-90594, US-865873 frag. ex CN!).
Uralepis brevicuspidata Buckley, Proc. Acad. Nat. Sci. Philadelphia 1862: 93-94. 1862. TYPE: U.S.A., Northern Texas, Wright 767 (lectotype: PH! designated by Hitchcock Man. Grass. U.S. 877. 1935 but without designating a herbarium; isolectotype: US-2786821-photo!).
Ipnum mendocinum Phil., Anales Univ. Chile 36: 211. 1870. *Diplachne mendocina* (Phil.) Kurtz, Bol. Acad Ci. (Córdoba) 15: 521. 1897. *Eragrosits mendocina* (Phil.) Jedwabn., Bot. Arch., 5(3–4): 192. 1924. TYPE: Argentina, Mendoza (holotype: SGO; isotype BAA-1452!).
Leptochloa pringlei Vasey ex Beal, Grass. N. Amer. 2: 436. 1896. *Diplachne pringlei* Vasey ex Beal, Grass. N. Amer. 2: 436. 1896. nom. inval. pro syn. *Leptochloa pringlei* Beal. TYPE: USA, Arizona, Pima Co., Arizona, Sierra Tucson, 27 Apr 1884, C.G. Pringle 13 (holotype: MSC-8177 seen digitally!; isotypes: GH, US-78806!, VT).
Diplachne dubia var. *kurtziana* Kuntze, Révis. Gen. Pl. 3 (2): 349. 1898. TYPE: Argentina, Córdoba, F. Kurtz 6647 (holotype: NY, fragment US-86574!).
Diplachne dubia var. *pringleana* Kuntze, Revis. Gen. Pl. 3 (2): 349. 1898. *Leptochloa dubia* var. *pringleana* (Kuntze) Scribn. & Merr., U.S.D.A. Div. Agrostol. Bull. 24: 27. 1901. TYPE: Mexico, Chihuahua, Hills and plains near Chihuahua, Pringle 422 (lectotype: NY! here designated, barcode 00019500; duplicates of lectotypes: GH!, MSC, NY-19498!, NY-19500!, P!, PH!, PR!, RSA!, US-899043!, VT).

#### Type.

MEXICO. F. Humboldt & A. Bonpland 4172 (lectotype: P! barcode P032678 designated by Snow et al., J. Bot. Res. Inst. Texas 2: 863. 2008; isolectotypes: B-Willd. barcode 02095-010! seen digitally May 2013, HAL-107044! seen digitally May 2013, K, K-microfiche, US-865876 frag. ex P!).

#### Description.

Perennials (or infrequently annuals; see below). Culms (5–)30–110 cm tall, 1.0–4.5 mm wide at base, round or flattened below, mostly erect or infrequently decumbent or sprawling, arising from fibrous roots, culms unbranched or only as tillers from very base; nodes glabrous; internodes 3–11 cm long, soft, solid or occasionally hollow with age. Leaf sheaths longer or shorter than internodes, sparsely pilose, especially below, and occasionally pilose (sometimes densely so) near the collar, the hairs occasionally with papillose bases, the margins glabrous or somewhat pilose; collars green or tan; ligules (0.5–)1.0–1.5 mm long, membranous, truncate, ciliate apically; blades (2–)8–35 cm long, 2–8 mm wide, cauline, mostly linear or somewhat narrowly ovate, flat but drying involute, scabrous above at base or sparsely pilose, glabrous to minutely scabrous below, midrib mostly prominent. Panicles of two types, the apical ones generally exserted at maturity and the lateral ones cleistogamous and completely hidden in lower leaf sheaths; apical panicles mostly 10–45 cm long, (2–)3−25 cm wide; branches (2–)5–15, (1.5–)3–19 cm long, alternate or infrequently subdigitate, ascending to reflexed, usually somewhat flexuous, minutely scabrous, the axils pilose or merely scabrous. Spikelets 4.0–12.0 mm long, most nearly sessile, imbricate to distant, 4–13-flowered; callus glabrous or with a few short hairs; lower glumes (1.6−)2.3–4.8 mm long, membranous, narrowly triangular or ovate, scabrous along midnerve, sometimes papillate on sides, acute; upper glumes 3.3–6.0 mm long, membranous, ovate to narrowly ovate, scabrous on midnerve (and sometimes with 1 or 2 additional nerves at least basally) and papillate on edges, acute; lemmas 3.5–5.0 mm long, 3-nerved (or infrequently 4– or 5–nerved above base), membranous, ovate to obovate or widely obovate, lateral nerves usually prominent but sometimes not so, sericeous at least along lower portions and sometimes on midnerve and between nerves, the hair tips rounded, apex usually bifid, broadly acute, obtuse, or truncate, awnless or mucronate; paleas membranous above and sometimes cartilaginous near base, subequal to lemma, narrowly ovate, distinctly ciliate on edges, sometimes sericeous between nerves, apex acute to obtuse. Anthers 1.0–1.6 mm long, yellow. Lodicules about 1 mm long. Caryopses 1.5–2.3 mm long, 0.7–1.0 mm wide.

**Leaf anatomy.** Previous authors discussed aspects of cross-section laminar anatomy of *Disakisperma dubium* (Brown 1960, Black and Mollenhauer 1971, Gutierrez et al. 1974, Valls 1978), whose collective data agree with our findings of the C_4_ NAD-ME photosynthetic pathway.

Midrib absent, or if present then lacking associated lacunae. Primary bundles (metaxlyem elements present) separated from one another by up to five secondary bundles (metaxylem elements absent). Primary and secondary bundles projecting little if at all adaxially or abaxially in fresh material. Outer primary bundle sheath cells continuous or interrupted adaxially but typically interrupted abaxially. Primary and secondary bundles often with colorless extension cells and sclerenchyma girders associated above and below. Colorless cells lacking between adjacent bundles; chlorenchyma always continuous. Bulliform cells present between primary and secondary bundles [Vouchers: *Snow 5865* (MO), *Snow 6673* (MO)].

**Stem anatomy.** The culm anatomy of *Disakisperma dubium* was discussed briefly by Canfield (1934) and Brown et al. (1959). The cross-section shown in Valls (1978, see fig. 21) is indented on one side where an axillary cleistogamous infloresence likely was located.

Culm solid. Outer sclerenchyma ring (subjacent to epidermis) present and sometimes surrounding outermost vascular bundles. Assimilatory (=assimilation) tissue (chloroplast-bearing and lightly staining parenchyma tissue (Pée-Laby 1898; Metcalfe 1960) often present between outermost vascular bundles. Additional vascular bundles scattered in outer ca. 1/3 of cortical tissue. Inner sclerenchyma ring absent or present (Valls 1978). [Voucher: *Snow 5865* (MO)].

**Chromosome number.** Individuals of *Disakisperma dubium* are tetraploid (Covas 1949; Valls 1978, voucher not seen), hexaploid (Brown 1950, voucher confirmed by Valls 1978), or octaploid (Gould 1960, 1965, vouchers confirmed by Valls 1978). Variation in ploidy number has not been shown to correspond with morphological variation or have a clear geographical pattern (Valls 1978), although sampling has been limited.

**Figure 2.**
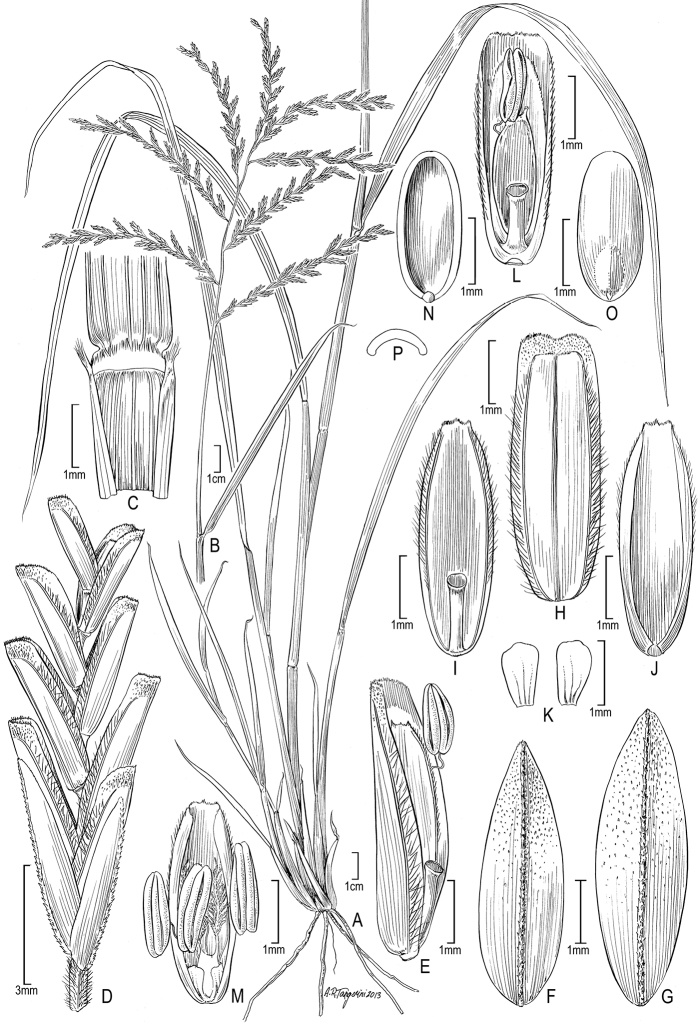
*Disakisperma dubium* (Kunth) P.M. Peterson & N. Snow **A** habit **B** culm andinflorescence**C** sheath, ligule, and blade, ventral view**D** spikelet **E** floret **F** lower glume **G** upper glume **H** lemma, dorsal view **I** palea, dorsal view **J** palea, ventral view **K** lodicules **L** floret, ventral view **M** perfect flower with lodicules, pistil, and stamens enclosed in palea **N** caryopsis, ventral view **O** caryopsis, dorsal view **P** caryopsis, cross section. A−C, M drawn from *Peterson & Annable 5387* (US); D−L, N−P drawn from *Peterson & Lara-Contreras 19890* (US).

#### Phenology.

Flowering commencing after the dormant season and continuing several months thereafter, or year–round in tropical climates at lower altitudes.

#### Distribution.

**Native:** In the USA from Arizona to Oklahoma and Texas, and disjunct in southern Florida, in much of Mexico, sporadically in the Carribean and in Mesoamerica to Bolivia and Chile east to Paraguay, Uruguay, and Argentina; in a variety of vegetation and soil types (including limestone), but most frequently on well-drained slopes, mostly 100–2500 m, to 3150 m in the Andes of South America (e.g., *Vargas 13770*[US]). (TDWG: AGE-CD, AGE-CN, AGW-JU, AGE-LP, AGW-CA, AGW-LR, AGW-ME, AGW-SA, AGW-SE, AGW-SJ, AGU-TU, BOL, CLM, ECU, MXC-DF, MXC-PU, MXE-AG, MXE-CO, MXE-CU, MXE-DU, MXE-GU, MXE-HI, MXE-NL, MXE-QU, MXE-SL, MXE-TA, MXE-ZA, MXN-BS, MXS-MI, MXT-CI, PER, PUE, ARI, FLA, NWM, OKL, TEX.) **Non-native:** USA in California, Hawaii (Snow and Davidse 2011), Kansas, and Missouri (TDWG: CAL, KAN, MSO). Its occurrences in the high Andes of Colombia, Ecuador, Bolivia, and Peru may be as introductions.

#### Conservation status.

Least Concern (IUCN 2010).

#### Etymology.

The Latin *dubium* means wavering or doubtful, which may refer to Kunth’s uncertainty about its inclusion in *Leptochloa* at the time of its description,but the intention behind the specific epithet is uncertain.

#### Vernacular names.

Green sprangletop; Texas crowfoot (Valls 1978), Zacate gigante (Beetle et al. 1991). Suggested name: American Jacobsgrass.

#### Comments.

The combination of its perennial habit, cleistogamous inflorescences inserted in the sheath near the base of the culms, short ciliate ligules, typically notched lemma apices, frequently (but not always) widely diverging florets during anthesis, and typically racemose to subdigitately arranged panicle branches, generally distinguish *Disakisperma dubium* from others in its geographical range. Among former components of *Leptochloa* s.l. (Snow 1997), *Disakisperma dubium* is sometimes confused with *Diplachne fusca* (L.) P. Beauv. ex Roem. & Schult. subsp. *uninervia* (J. Presl) P.M. Peterson & N. Snow, which also has truncate to (usually) emarginate lemma apices. However, *Diplachne fusca* subsp. *uninervia* is an annual (that sometimes is weakly perennial), has much longer and apically attenuated (and often lacerated) ligules, lacks hairs on the outer edges of the collar, lacks cleistogamous inflorescences, and grows most frequently in disturbed areas in heavier and more poorly-drained soils, often in disturbed habits or along watercourses.

The root hair formula of *Disakisperma dubium* is P-I-I (Row and Reeder 1957), whereas its embryo formula (Reeder 1957) is P+PP (Valls 1978). Branching, if present, usually occurs only at the basal most nodes (Valls 1978). The cleistogamous lateral inflorescences (Parodi 1927; Gould 1975; Valls 1978), which correspond to subtype 1b of Campbell et al. (1983), do not occur in the *Disakisperma eleusine*, *Disakisperma obtusiflorum*, or *Disakisperma yemenicum*. Obligate cleistogamy by means of inserted inflorescences in Eragrostideae (sensu Clayton and Renvoize 1986) also occurs in some species of *Muhlenbergia* Schreb., *Triplasis* P. Beauv., and *Cleistogenes* Keng (Clayton and Renvoize 1986; Peterson and Annable 1991; Chen and Phillips 2006). Species formerly placed in *Leptochloa* s.l. (Snow 1997) in which basal panicle branches typically are not completely exserted, and for which some degree of cleistogamy likely occurs, include *Dinebra viscida*, *Diplachne fusca* subsp. *fascicularis* (Lam.) P.M. Peterson & N. Snow,and *Diplachne fusca* subsp. *muelleri* (Benth.) P.M. Peterson & N. Snow.

*Disakisperma dubium* exhibits considerable morphological variation across its range, which is expected given its wide geographical distribution. Some populations of *Disakisperma dubium* are somewhat distinct locally and might be found to be genetically distinct. For example, the life cycle of some specimens from Baja California is annual (Valls 1978; e.g., *Rebman 7548 et al*. and *Dominguez L. 3062* (both at ARIZ)). In the Baja and Sonora states of Mexico some specimens [e.g., *Gould 12183* (K, TAES), *Felger et al*. *92–966* (MEXU), *Carter & Moran 5317* (GH), *Carter 4781* (GH)] are atypical in having some combination of the following characters: palea with appressed hairs between the nerves, caryopses widely to very widely ovate, and lemmas obovate to widely obovate with very wide apical lobes (Valls 1978). Many specimens from Kenedy and Cameron counties in south Texas, an area where sandy soils are common, are characterized by relatively thin, short, densely cespitose culms having short, few-branched panicles, an overall growth form that also occurs in some plants from Argentina. The short stature, however, may be a phenotypic response to frequent grazing by cattle (Valls 1978) or due to lower levels of soil moisture typical of sands. Variation in lemmatal shape and spikelet shape also can be considerable (Valls 1978; Nicora 1995; Snow 1997, 2012). For example in Argentina, where the species is cultivated (Nicora 2006), specimens may have either exceptionally wide lemmas or acute, unnotched lemmatal apices. The lemmas and upper glumes occasionally have indistinct additional nerves at the base. The palea is atypical and somewhat cartilaginous and flared outwards near the base, thereby surrounding the adjacent rachilla segment. Since morphological variantion intergrades more or less continuously, we feel that recognition of infraspecific taxa is unwarranted.

Snow (1997) included *Leptochloa digitatiformis* Beetle (1982) as a synonym of *Disakisperma dubium*, which merits additional explanation. The original description by Beetle (1982) was sparse, who for reasons unknown compared the species to *Leptochloa chloridiformis*, a non-persisting South American species collected for a brief period in North America during the 1940s in Cameron County, Texas (Snow 2003). Beetle’s concept of *Leptochloa digitatiformis* was a combination of *Disakisperma dubium* and *Chloris submutica* Kunth, the latter of which is sometimes perceived as immature *Disakisperma dubium* and misidentified as such. The basis for this conclusion is a result of the first author having seen numerous specimens of *Disakisperma dubium* and *Chloris submutica* annotated in Beetle’s hand as *Leptochloa digitatiformis* (e.g., *White 3606* [MEXU], *Cervera 90* [SARH], *Carranco & Brito 59* [SARH], *Murrieta 17* [SARH], *Banks 1832* [WYAC], *Llmeida et al*. *121* [WYAC]). This name is now exluded (see Excluded name, below) from *Disakipserma*.

The species apparently provides moderate forage quality for livestock (Valls 1978; Nicora 2006) although it rarely occurs in dense stands in native vegetation. Marone et al. (1998) reported that the caryopses of *Disakisperma dubium* constitute a significant component of the seed mass consumed by graniverous birds in the Monte Desert of Argentina.

Valls (1978: 144) reported thrips (Thysanoptera) in the spikelets of *Disakisperma dubium*.

#### Specimens examined.

Argentina. Catamarca: Dpto. Belén, Laguna Blanca, Cabrera et al. 32526 (SI); Dpto. Belén, Quebrada del Belén, Cabrera et al. 16758 (LP); Dpto. Andalgala, Jörgensen 1352 (GH, MO, NY, UC, US); W base of Cuesta La Chilca, 14 road km E of Andalgala jct, Hwy 46 on Hwy 365/48, Peterson, Soreng, Solariato & Panizza 19418(US); Sierra de Belén, NW of Condor Huasi, ca. 14 air km NW of La Puerta de San Jose, jct Hwy 40 N of Belén Peterson, Soreng, Solariato & Panizza 19407 (K, US); Dpto. Belén, Las Mansas, Schreiter 698 (GH); Valle Catamarca, Parodi 13983 (BAA); Balcones, Sierra Ambato, Parodi 14072 (BAA): Dpto. Andalgalá, El Ingenio, Cabrera et al. 24723 (LP); Dpto. Belén, Quebrada de Belén, Cabrera et al. 23773 (LP). Dpto. Paclin, 19 km E of Catamarca on Hwy 42 towards Puesto del Portezuelo, Peterson & Annable 11641(AAU, GH, K, MO, P, NY, RSA, TAES, UC, US, UTC). Córdoba: Plaz Colón, Kurtz 8866 (NY); Bei Córdoba in Argentinien, Stuckert 378(PR); Dpto. Capital, Quinta en los alrededores de la Ciudad de Córdoba, Stuckert 11187 (MO, NY); Dpto. de San Javier, San Javier, Bridarolli 1270 (LP); Camino de Casilla del Monte a San Marcos, Nicora 2435 (MICH); Valle de los Reartes, Parodi 166 (BAA); Capilla de Remedios, Parodi 6476 (BAA); Ciudad de Córdoba, Dpto. Capital, Stuckert 5725 (MO, NY, US). Corrientes: Dpto. Prim. de Mayo, Colonia Benítez, Schulz 17291 (SI). Jujuy: On open rocky Mt. slope, Alfarcito, E of Tilcara, Correll et al. A669 (US); Dpto. Tumbaya, El Moreno, Cabrera et al. 22441 (LP); Dpto. Tilcara, Pucara, Cabrera et al. 23553 (LP); Dpto. Tumbaya, camino de Purmamarca a Abra de Pives, Cabrera 18523 (LP); Volcán, Cabrera & Frangi 20645 (LP). Dpto. Humahuaca, Azul Pampa, Cabrera et al. 21410 (LP); Dpto. Purmamarca, subida a Tascal, Cabrera et al. 15082 (LP); Maimará, Sierra de Zenta, Budin 1510 (BAA). La Pampa: Dpto. Sierra Lihuel-Calel, Sierra Lihuel-Calel, Peterson & Annable 11233 (K, MO, US). La Rioja: Dpto. Famatina, Los Corrales, Cabrera et al. 27223 (SI). Dpto. General Lavalle, Sierra de Sanogasta, W side of Cuesta de Miranda ca. 20 km W of Miranda on Hwy 40, Peterson & Annable 11566(K, MO, RSA, US). Dpto. Rosario Vera Peñaloza, Chelcos, Stuckert 18788 (MO, NY); Sierra Velazco, Morello 5087 (LP); Dpto. Chilecito, Guanchin, Cabrera et al.24626 (LP); Puerta de Miranda, Parodi 7837 (BAA); Sierra de Famatina, Camino a La Mejicana, Parodi s.n. (BAA); Sierra de Sanogasta, 43 air km due E of jct Villa Union, jct with Hwy 76, 1 road km W of Cuesta Miranda, Peterson, Soreng, Solariato & Panizza 19332 (GH, K, MO, NY, RSA, US, UTC). Mendoza: Dpto. Las Heras, Capdevila, Covas 3593 (SI); Prov. de La Rioja, Ruiz Leal 16724 (ARIZ);10 km N of Uspallata on Hwy 39 towards Calingasta, Peterson & Annable 11456(K, MO, RSA, US); 25 km N of Upsallata on road towards Calingasta, Peterson & Annable 11472(GH, K, MO, NY, RSA, US, UTC). Dpto. Santa Rosa, Nicora et al. 8367 (MO, SI); Dpto. Godoy Cruz, Cacheuta, O’Donell 1115 (MO); Villavicencis, Beetle 699 (GA); Cerro de la Gloria, Contardi 13 (LP); Dpto. Lujan, Dist. Chacras de Coria, Bartlett 19195 (MICH); Dpto. San Rafael, San Rafael, Otamendi 17184 (BAA); San Rafael, Schulz 6183 (LP); 35 km SW of San Rafael on Hwy 144 and 4 km W of road, Peterson & Annable 11347(K, MO, NY, US). Dpto. Tupungato, N of Rio de la Carrara on Hwy 89 at jct to Estancia La Carrera, 6.3 road km NW of San Jose, jct. Hwy 86, Peterson, Soreng, Solariato & Panizza 19240 (K, MO, NY, RSA, US). Salta: Dpto. Santa Victoria, Santa Victoria, Meyer 4999 (NY, UC); Dpto. Poma, Canguejillos, Cabrera 8826 (LP); Nevada de Cachi, 15 km NW of Cachi just below the Ruinas Las Pailas, Peterson, Annable & Morrone 10192 (K, MO, US); Valles Calchaquies, ca 66 air km N of Cachi, Hwy 40, 68 rd km N of jct of Hwy 38, N of La Quesera, S of Abra del Acay, Peterson, Soreng, Solariato & Panizza 19511 (GH, K, MO, NY, RSA, US, UTC). Dpto. Tupungato, 3 km NW of San Jose on Hwy towards La Carrera, Peterson & Annable 11391(K, MO, NY, RSA, US); Dpto. La Poma: Malpaso, ca. 25 km E of Súsques along rte 40, Taylor et al. 11243(MO, US). San Juan: Dpto. Zonda, Camino a Estancia Maradona, Kiesling 4348 (SI); Dpto. Ullún, Hualilán, Kiesling 7858 (SI); A Carpineria on dry sterile banks in rocky hills at 2500 m in central Andes, Beetle 657 (GA); 48 mi E of Cachi on Hwy 40 to Salta and 2 km W of turnoff to Amblayo and Iszona, Peterson & Annable 10213 (K, MO, RSA, US); 40 mi SW of Zonda at Agua Pinto (Estación Maradona), Peterson & Annable 11505(K, MO, NY, P, RSA, US). Dpto. Iglesia, 25 km W of Las Flores on Hwy 150 towards Puerto del Agua Negra, Peterson & Annable 11538(K, US); Valle de Pismanta, along Hwy 150 to Porto del Agua Negra, 16 road km W of jct Hwy 150 in Las Flores, Peterson, Soreng, Solariato & Panizza 19279 (K, MO, NY, RSA, US). Santiago del Estero: Dpto. Guasayán, Ea. “El Mangrullo”, Kunst & Perez4 (UC). Tucumán: Dpto. Tafi, Los Sauces, Peirano 276 (NY); Dpto. Grancas, Vipos, Venturi 1675 (GH, US); Tafi del Valle, Region Montañosa entre San Javier, cumbres calchaquies y Amaicha (Valle Calchaqui), Parodi 10978 (BAA); Dpto. Tafi, Tafi del Valle, Türpe 704 (M); Ca. 12 km W of Amaicha del Valle on Hwy 307 towards Tafi del Valle, Peterson & Annable 11613(K, MO, US); Tapia, Venturi 2338 (US). Bolivia. Campero: Cochabamba, localidad alreadedores de Pasorapa, Saravia 659 (MO); Cochabamba, Parodi 10199 (BAA); La Paz, Murillo (Valencia/Mecapaca); Stony slopes above the village, Renvoize & Cope 4234 (US); La Paz, Murillo, Mecapaca, Rocky slope above village, Renvoize & Cope 4240 (MO, US). Potosí: Approximately 8 km S of Tupiza on Hwy 702 towards Villazon, Peterson & Annable 11860(US). Tarija: Ab. loco, Fries 1094(US). Columbia. Nariño: Río Guaitara valley near jct of Pan Americana and road to Tuquerres, Wood 5326(COL, K, US). Mun. Imúes, corregimiento El Pedregal, Pilcuán, B. Ramírez 1259 (COL, PSO). Carretera Pasto-Túquerres, C. Saravia & R. Jaramillo 1869 (COL). Ecuador.Azuay: 11 km N of Ona on the Pan Am. Hwy near the Río Leon, Peterson, Annable & Poston 8907 (MO, US); 80 km SW of Cuenca on road to Loja, 3 km N of Río León, Peterson & Judziewicz 9374 (US). Loja: Km 13.5 Catamayo-Catacocha, Lægarrd & Kullberg 71178 (NY). Pinchicha: El Pisqaue, Acosta Solis 16300(US). Mexico. Aguascalientes: 11 mi N of Rincon de Romos, Shreve 9246 (ARIZ); 7 mi S of Aguascalientes, Soderstrom 712 (ARIZ); 6.4 m E of Aquascalientes on MEX Hwy 70 to San Luis Potosi, Peterson 9672 (BISH, K, MO, US); Hwy to Ojuelos, Jal., 9 mi E of Aguascalientes, McVaugh 16641 (NY); Mpio. Palo Alto, Mesa de Preñadas, Palo Alto, Gutiérrez 89 (ENCB). Baja California Sur: Ca. 2 mi E of Route 1, along road to San Basilio, between Loreto and Mulege, Rebman 7548 et al. (ARIZ, ASU); Aguaje Los Tules, San Jaun de la Costa Municipio de La Paz, Dominguez L. 3062 (ARIZ); One mi SW of Jct of road to Huatomote along Hwy Mex. 1, Reeder & Reeder 6717 (ARIZ); 54 mi NW of La Paz, Gould 12200 (US); 7 mi W of Loreto on road to Las Parras, Reder & Reeder 6649 (RM); Cape St. Lucas, Xanthus 119 (GH); Isla del Carmen, lado W de la isla, Puerto Balandra, a 100 m de la playa, Sousa 136 (MO); 15 mi SE of San Antonio, Gould 12159 (TAES); Cañon del Cayuco, E base of Cerro de la Giganta, Carter & Kellogg 3107 (ARIZ, GH, LL, UC, US); S of La Paz, km 100 near San Bartolo, Beetle M–2561 (NA, RSA, TAES); Mts. E of Loreto, Jones 27624 (POM); 14 mi E of San Ignacio, Wiggins 11370 (GH, POM, UC); 3 mi SW of San Pedro on La Paz-Todos Santos Rd., Gould 12183 (MICH, TAES, TEX, UC, US); 5 mi W of Loreto, BeetleM–2428 (TAES); Peaks south of Portezuelo de la Cuesta de los Dolores, Sierra de la Giganta, Carter 4781 (ARIZ, GH, MEXU, MICH, MO, TAES, UC); Mouth of Cañada del Aguaje, Valle de Los Encinos, S side of Cerro Giganta, Sierra de la Giganta, Carter & Moran 5317 (GH, MICH, TAES, UC); Just W of summit peak of Mesa San Gerónimo, northerly from Rancho Viejo, Carter 5028 (TAES, UC). Chiapas: Mpio. San Cristóbal, NE edge of San Cristóbal Las Casa, Breedlove 51440 (CAS, MO, NY). Chihuahua: Km 1918 carretera Cd. Juarez, Hernandex & Mathus N-1906 (RM);50 km SW of Coyame of Mex 16 between Presidio, Texas and Chihuahua, Peterson & Annable 5761(US); Nuevo Majalca; Ca. 14.5 km W of HWY 45, N of Chihuahua, Peterson and Annable 5769(US); 58 km N of Parral on MEX 24 towards Chihuahua, Peterson & Annable 8095(BISH, US); 87.7 km N of Parral on MEX 24 towards Chihuahua, Peterson & Annable 8105(BISH, K, MO, US); Sierra El Nido, 6.1 mi W of Hwy 45 on dirt road towards Santa Clara, Peterson & Annable 12581 (US); 10 mi N of Ciudad Camargo, Reeder & Reeder 2612 (MEXU); Sierra Madre Occidental, 11 mi SE of Balleza on road to Parral, Peterson, Knowles, Deitrich & Braxton 13525(K, US); Sandstone hill 8 mi NW of Cruces, Shreve 8911 (MICH); Km 11 entre San Buenaventura y El Carmen, carretera Nuevo Casas Grandes, Hernández & Mathus N–1940 (GH); 7 mi NE of Janos at top of Mt. with radio tower, Luckow et al. 13239 (TEX); 7.4 km W of Balleza on road to Guachochi, Peterson, Annable & Valdes-Reyna 10727 (ANSM, BISH, US); Ca. 31 (air) mi NW of Julimes in a SW-facing canyon above Rancho El Recuerdo in Sierra de Carrasco, Henrickson 12924 (LL); 17 (road) mi NE of Aldama along Hwy 16 at Puerto de Gomez, Henrickson 7544 (LL); 13.3 (road) mi NE of Aldama along Chih. Hwy 16, Henrickson 7524 (LL); Canyon La Campana, near Encinillas, Knobloch 613 (TAES); Along Chihuahua Hwy 0, ca. 57 mi S of Mexico Hwy 16 at Ojinaga and ca. 40 mi N of turnoff to La Perla, and 30.9 mi S of Lan Mula, Reveal & Atwood 3310 (NY, TEX); W of Buenaventura along Hwy 28, 7.4 mi W of jct Hwy 10 in Buenaventura, Mayfield et al. 114 (ARIZ, TEX); Hills and plains near Chihuahua, Pringle 422 (GH, NA, NY, P, RSA, US); Carretas, White 1115 (ARIZ, MICH); Near boundary with Durango at 10 mi SW of El Ojito, Peterson Sánchez-Alvarado & Gómez-Ruíz 20026 (BISH, K, MO, US); Santa Eulalia, Velardeña arroyo, Hewitt 238 (GH); Mpio. Hidalgo del Parral, 10 km al sur de Parral, Blanco-Aguirre 2108 (ENCB); Rancho Carretas, Harvey 1620 (MO, US); Ca. 8 mi S of Ixmiquilpan, Reeder 1615 et al. (ARIZ). Coahuila: Saltillo, Hitchcock 5631 (RM); 12 mi S of Saltillo, Reeder & Reeder 2912 (ARIZ);5 mi S of Castanos, Reeder 3284 et al. (ARIZ); 9 km S of Parras on Sierras Negras, Standord 2400 et al. (ARIZ); 30 km al Pte. de G. Cepeda, carretera a Parras, Valdés VR-1577 et al. (ARIZ); N side of Canon de la Fragua, ca. 25 mi SW of town of Cuatro Cienegas on Mexico Hwy 30, Van Devender 84-601 et al. (ARIZ); 12 mi W of San Buenaventura, Reeder & Reeder 3932 (ARIZ); Approximately 32.2 km SE of Saltillo on road to Los Lirios, Peterson & Annable 6248(US); ca. 6 km S of Saltillo, owned by Univer. Autónoma Agraria “Antonia Narro”, Peterson & Valdes-Reyna 8341(BISH, K, MO, US); 85.4 km NW of Muzquiz, on Hwy 53 towards Boquilla del Carmen, via La Rosita, Peterson & Annable 10573(BISH, US); Madera del Carmen; 9.7 mi NE of Las Pilares, Peterson, Saarela, Lara Contreras & Reyna Alvarez 20940 (ANSM, US); N of Los Pilares, Peterson & Romaschenko 24472 (ANSM, US); Madera del Carmen, road between Campo Cinco and Campo Uno, Peterson & Romaschenko 24554 (ANSM, US); 135.4 km NW of Muzquiz, on Hwy 53 towards Boquilla del Carmen, Peterson & Annable 10580(BISH, GH, K, MO, NY, RSA, US); Sierra El Pino, 18.8 km SW of Rancho El Cimarron, Peterson & Annable 10646(BISH, K, MO, US); Sierra Zapalinamé, along camino “El Cuartro”, E of Saltillo, Peterson & Valdes-Reyna 18803 (BISH, K, MO, US); Sierra del Carmen, Ejido San Francisco, near small cabin, Peterson, Valdes-Reyna & Sifuentes 18823 (BISH, K, MO, US); Sierra El Jardin, Peterson & Lara-Contreras19918 (K, MO, US); Sierra El Jardin, slopes above Canyon del Diablo, Peterson & Lara-Contreras 19890 (BISH, GH, K, MO, NY, RSA, US, UTC); Sierra El Jardin, Canyon del Diablo, at 8 miles from jct of road towards Boquillas, Peterson & Lara-Contreras 19871 (K, MO, US); 99.6 mi N of Melchor Muzquiz at Cuesta de Malena on Hwy 25 towards Boquillas del Carmen, Peterson & Laras-Contreras 19832 (US); Cañon de Madera, W side of Sierra de los Guajes, ca. 4 km E of Rancho Buena Vista, Stewart 1506 (GH, US); Ca. 18 (air) mi NE of Tlahualilo, 9 (rd) mi NW of Los Charcos de Risa, Henrickson 13698 (ARIZ); Cuesta Zozaya, ca. 38 km de Ocampo rumbo a Sierra Mojada, Carranza & Carranza C–680 (MO); Sierra de la Madera, vicinity of “La Cueva”, Johnston 9103 (GH, MO, US); Sierra de la Paila (Lado Norte) Cañada becerros, Villareal et al. 5449 (ANSM); 11 km W of Saltillo, Mick & Roe 24 (TAES); Rancho “El Porvenir”, located N of “La Bahia”, Huss39–69 (TAES); 10 mi SE of Saltillo on old road to Argeaga, Gould 8691 (TAES, UC); Sierra de la Gavia, Valdés–Reyna et al. 1710 (TEX); 4.6 mi W of Rancho Ganadero,Los Angeles, Peterson, Saarela, K Romaschenko & Valdés-Reyna 23120 (ANSM, US); Buenavista, a 6 km al sur de Saltillo por la carretera Saltillo-Zacatecas, carr. 54, en Universidad A. A. “Antonio Narro”, Snow& Valdés-Reyna 6675 (ANSM, MEXU, MO); Entroque Derramadero, a 20 km al Sur de Saltillo por la carretera Saltillo–Zacatecas, carr. 54, Snow & Valdés-Reyna 6696 (ANSM, MEXU, MO); Entrada de la Carretera 54 a Rancho Los Angeles, 52 km al Sur de Saltillo, Snow & Valdés-Reyna 6710 (ANSM, MEXU, MO); Sierra de Arteaga, Rancho “El Chorro”, Carretera 57, casi 0.5 km pasando el entronque a Los Lirios, cerca de encampamiento “El Chorro”, Snow & Carranza 6764 (ANSM, MEXU, MO); Mpio. de Arteaga, Sierra de Arteaga, Bella Union, ca. 2 km al E de Arteaga, Carretera 57, Snow & Carranza 6765 (ANSM, MEXU, MO); 1 km E of San Antonio, Mpio. Arteaga, Peterson & Romaschenko 24566 (ANSM, US); Santa Rosa Mts., Marsh 1449 (TEX); Norias de Guadalupe, 100 km al SW de Acuña, Cabral 954 (TEX); Sierra de los Organos, Wendt & Lott 1396 (LL); 9 km S of Parras on Sierras Negras, Stanford et al. 189 (GH, MO, NY, UC); Del Carmen Mts., Marsh 723 (GH, TEX); Cuatro Ciénegas, Puerto del norte, Harvey 1226 (MICH); 2 mi W of Saltillo, road to Torreón, Harvey 1092a (GH, MICH, MO,US); Serranias del Burro Mts., Rancho Margareta headquarters, ca. 65 mi NW of Sabinas, Gould 10623 (MICH, TAES, UC); La Escuela Superior de Agricultura, Buena Vista, ca. 5 mi SE of Saltillo, Gould 6387 (MICH, RSA, TAES, TEX, UC); 1 mi SE of San Antonio de las Alazanas (SE of Saltillo), Gould & Watson 10516 (MICH, TAES, TEX, UC, US); Sierra de las Cruces, eastern foothills 8 mi N of Santa Elena Mines, Johnston & Muller 1029 (GH, LL, US); Sierra del Pino, vicinity of La Noria, Johnston & Muller 493 (GH, LL, NA); Vicinity of La Noria, Steward 1201 (GH, TEX, US); 13 km N of Rancho El Jardin on winding road to Mina El Popo, Johnston et al. 11854A (LL); La Cuesta del Plomo on the Músquiz–Boquillas Hwy, Johnston et al. 9199 (LL, MEXU); Mina El Popo, ca. 2 km S of Cañon del Diablo on dissected E slope of Sierra del Carmen, ca. 19 km by winding road N of Rancho El Jardin, Johnston et al. 11912 (LL, MO, NY); 3.7 (road) mi S of Parras, Henrickson 6177b (LL); Ca. 45 (air) mi W of Cuatro Cienegas, 4 mi SW of Hacienda Zacatosa, Henrickson 12056 (LL); Ca. 35 (air) mi SSW of Cuatras Cienegas in northern slope of limestone Sierra de Los Alamitos, ca. 9.2 road mi S of El Hundido, in Izotal, Henrickson 13677b (ARIZ, LL); Ca. 12 (air) mi E of Boquillas, Henrickson 11553 (LL); Ca. 27 (air) mi SE of Torreon in Sierra de Jimulco, ca. 6 (air) mi SSW of La Rosita, ca. 1/2 mi along rail beyond road’s end, Henrickson 13235 (LL); Ca. 18 (air) mi NE of Tlahualilo, 9 road mi NW of Los Charcos de Risa, Henrickson 13698 (LL); Ca. 54 (air) mi SE of Big Bend N.P. Basin in S end of Sierra Maderas del Carmen in the Cañon de la Fronteriza, 2 mi NE of Rancho San Isidro, Henrickson & Prigge 15046 (LL); Serranias del Burro, Rancho El Bonito, Mpio. de Villa Acuna, Riskind et al. 2187 (ANSM, TEX); 15 mi W of Saltillo on Hwy to Torreon, Gould 11556 (TAES); 0.5 mi N of Las Vacas, Ely 156 (TAES); Cañon del Agua, 1.3 mi S of ranchito, Valdés-Reyna & Wendt 1016 (TAES); S end of Puerto San Lázaro (Cuesta La Muralla), 0.5 mi N of San Lázaro along Rte. 57, Valdés-Reyna & Wendt 1106 (ANSM); Sierra de Zapaliname Mts., 5 km S of Saltillo, Hatch et al. 4489 (TAES); 53 km S of Saltillo along Hwy 54, S of Concepcion del Oro, Hatch et al. 5412 (TAES). Distrito Federal: Pedregal de San Angel, 1 km S of UNAM, Nee 238 (MEXU); Sierra de Santa Catarina, parte alta Delegación de Ixtapalpa, Rzedowski 33862 (ANSM); San Angel, Orcutt 3691 (GH, MICH, MO, NY, TEX); Sierra de Guadalupe, Balls 5612 (MICH, UC); Tacubaga, St. Pierre 872 (MICH, US); Del. Coyoacan: Pedregal de San Angel, Sharp & Gilly 180 (MICH); Del. Ixtapalapa, Partes altas del Cerro Estrella, Koch 77203 (ENCB, TAES, US); Pedregal de San Angel, Fernández 9 (ENCB, TEX); Lomas, Lyonnet 1735 (CM, ENCB, MEXU, MO, UC, US); Around Coyoacan, Antipovitch 40 (CM); Vicinity of México, Hitchcock 5892 (US); Mixcoac, Olivar, Arsene 8283 (GH, US); Mixcoac, St. Pierre 2250 (MICH); Sierra de Santa Catarina, parte alta, Del. de Ixtapalapa, Rzedowski 33862 (ENCB); San Gregorio, Del. de Xochimilco, Ventura 2105 (ENCB, MEXU, TAES); Sierra de Guadalupe, Ticomán, Bopp 38 (ENCB). Durango:35.5 km N of Durango on MEX Hwy 45, Peterson 9647(BISH, K, MO, US); A 11 km de Nombre de Dios, sobre la carretera a Durango, Herrera 692 (MEXU, NY); Ca. 18 km al E de Durango, carretera a México, Herrera 438 (MEXU); Hwy 49, 1.5 mi N of Rancho Grande, ca. 15–20 mi S of Rio Grande, Spellenberg 2935 (NMC, NY); Ca. 18 km al E de Durango, carretera a México; Herra 438 (ANSM); 1.3 mi SE of San Jose del Molino, Peterson, Saarela, Rosen & Reid 21170 (CIIDIR, US); Estacion Microondas “Sapioris” ca. 30 km SW of Gomez Palacio on Hwy toward Durango, Johnston et al. 10399 (LL, NY); 3 mi N of Donato Guerro, Emery 342 (TEX); ca. 35 mi N of Rodeo, Emery 357 (TEX); Mpio. Súchil, al SE de Súchil, González 2023 (TEX); Mpio. Mapini, Top of Perto de la Cadena, Sanders et al. 6689 (ARIZ, RSA); Santiago Papasquiaro, Palmer 468 (BAA, MICH, MO, UC, US). Guanajuato: 79 mi N of Queretero (1 mi S of San Luís Potosí state line), Waterfall 16563 (UC); About 5 mi E of Silao along the road to Guanajuato, Reeder & Reeder*2287* (GH); 5 km E of Jofre, Peterson, Saarela & Romaschenko 23422 (CIIDIR, US); 10 km al N de León, Mpio. León, Galván & Galván 3039 (MO); Rancho Las Adjuntas, Mpio. de San José Iturbide, Ventura & López 9473 (ANSM); La Angelita, Beetle et al. M–1723 (NA, TAES); Cerro Capulin, just E of Mexico 43, ca 0.8 km NNE of Vriangato on road to Salamanca, Iltis & Doebly 147 (TAES); 20 mi NW of Irapuato, Barkley et al. 764 (TEX); Mpio. Jerequerro, near Fresno, Beetle M–7166 (RSA); 39 km al SW de Cuerámaro, sobre el camino a la Barranca del Chilar, Rzedowski 47214 (ENCB); Ca. 22 mi NE of San Luis de la Paz, Reeder & Reeder 2257 (GH); Mpio. San Luis de la Paz, El llano 1 km de la comunidad de San José de jofre, González s.n. (ENCB); Mpio. Santiago Papasquiaro, grounds of Escuela José Ramon Valdéz, Díaz 611 (TAES). Hidalgo: Cerro Ventoso, entre Pachuca y Real del Monte, Rzedowski 20578 (GREE, MEXU); Pachuca, Orcutt 3917 (MO, US); 10 km al NNW de Ixmiquilpan, Gonzalez Quintero 2735 (RM); 7 km al NNE de Tasquillo, González 2966 (TAES); Tailings dam from Loreto Mill, Santa Julia near Venta Prieta, Dsto. Pachuca, Moore 3165 (GH, US); 8 km al SSW de Alfajayacun, González 3027 (TAES); Durango, Mpio. Hidalgo, El portento (Rancho las Iglesias), Ríos 12 (TAES); Ca. 8 mi S of Ixmiquilpan, Reeder et al. 1615 (RSA); 8 mi S of Ixmiquilpan, Gould 9302 (MICH, TAES); 11 mi N of Ixmilquipan, Gould 9566 (TAES); Cerca de los pitos, Matuda 21519 (MEXU, MICH); Cerca de los pitos, Matuda 21496 (MEXU, MO, TEX); Cerro Ventoso, entre Pachuca y Real de Monte, Rzedowski 20561 (ENCB, NY, US); Mpio. Zempoala, Sierra de los Pitos, Tlaquilpan, Benítez 122 (ENCB). Jalisco. Lagos de Moreno, Beetle M-5623 & Guzman M. (ARIZ); El Jalocotal, 2-3 km W de El Jalocote, Santana 8729 (BRIT); Barranca of Río Verde, ca 20 mi N of Tepatitlán on road to Yahualica, McVaugh 17431 (NY); 7 mi SE of the junction of Hwys 80 and 45 at Lagos de Moreno, Davidse & Davidse 9937 (MO); 11 mi N of Ciudad Guzman, Gould 9638 (TAES, TEX); 48 mi SW of Aguascalientes on Hwy 45, Pratt 630 (TEX); 10 mi E of Tepatitlan, Gould 9653 (TAES, TEX, UC); 8 mi NE of Lagos de Moreno, Gould 9657 (TAES); Cerro de los Gallos (ca. 15 mi S of Aguascalientes, McVaugh 17126 (TAES); Barranca of Río Verde, ca. 20 mi N of Tepatitlán on road to Yahualica, McVaugh 17431 (TAES). Mexcio D.F.: Mpio. Texcoco, Poblado de Huexotla, Teresea Licona 14 (MO); Old Hwy 190 between turnoff to Chalco (Hwy 115) and Santa Barbara ca. 30 mi above Azotla, Mick & Roe 288 (TAES); Mpio. Ajapusco, Cerro de Jaltepec, Ventura 168 (FLAS, MEXU); Mpio. Ajapusco, Terrenos de Jaltepec, Ventura 1997 (ANSM, TAES); Satilite (NW edge of Mexico City on Hwy 57), Gould 10190 (TAES); San Vicente Chicoloapan, Mpio. Texcoco, Ventura 4103 (TAES); 16 mi N of Cuautitlan, Gould 10205 (TAES, TEX); Huehuetoca, Matuda 26647 (MEXU, MO, TEX); Cercanías de Tultepec, Matuda et al. 29336 (MEXU, MICH, NY); Pegregal de Tlalpan, Matuda 21376 (MEXU, MICH, MO); Mpio. Huehuetoca, Cerro Mesa La Ahumada ladera Oeste, Romero–Rojas 1612 (ENCB); Mpio de Temascalpa, 1 km al oeste de Temascalpa, Espinosa 786 (ENCB, MEXU); Cerro Buenavista, Temascalapa, Castilla & Tejero 765 (ENCB); Mpio. de Axapusco, 12 km al NE de San Martín de las Piramides, por la carretera a Tulancingo, Hgo, Koch 77170 (ENCB, TAES, US); San Juan Teotihuacan, Fisher s.n. (CM). Michoacán: Ca. 1.7 mi NW of Tuxpan, near km post 129 on Hwy 15 to Morelia, Davidse & Davidse 9816 (MO); 4 km E of San José Coapa along MEX 14, Steinmann & Steinmann 1933 (ARIZ); Cerro del Bao, cerca de Tzurumutaro, Mpio. Pátzcuaro, Escobedo 1668 (ANSM); 2 km al N de Catedral Porvenir, Mpio. Tarímbaro, Zamudio 4413 (ANSM); Hills of Lake Cuitzeo, on road from Morelia to Cuitzeo del Porvenir, Sohns 741 (TAES, US); Ca. 5 mi SW of Quiroga, Barkley et al 2725 (TEX); 6 mi E of Ciudad Hidalgo on Hwy 15, Pratt 741 (TEX). Nuevo Leon: 1 mi E of San Marcos, ca. 13 mi SE of Galeana, Gould 10725 (MICH); Mpio. Galeana, Chase 7742 (ARIZ); Along the big arroyo W of Galeana, Hwy no. 60, Cummins s.n. (RM); Monterey, Tateoka 1100 (TAES); Sierra Madre Oriental, El Salero, Peterson and Valdes-Reyna 15817 (BISH, US); Sierra Madre Oriental, 6.5 mi S of Border of Coahuila and Nuevo Leon on Hwy 57 towards Matehuala, Peterson & Knowles 13285(BISH, K, MO, RSA, US); 5.2 mi S of Zaragoza on road towards Ejido La Encantada, Peterson, Valdes-Reyna & Sosa-Morales 16752(US); 9.4 mi W of San Antonia de Peña Nevada and 0.4 mi E of Jtn of Hwy 2 to Or, Peterson, Valdes-Reyna & Sosa-Morales 16788(US): 4.3 mi E of Hwy 57 on Mex 31 towards Linares, Peterson, Saarela & Romaschenko 23182 (US); 0.1 mi W of Once de Marz, Peterson, Saarela & Romaschenko 23611 (CIIDIR, US); 14.4 mi S of Casas Blancas, Peterson, Saarela & Romaschenko 23686 (CIIDIR, US); 5 km E of San Roberto on Hwy 62 towards Galeana, Peterson, Romaschenko & Valdés-Reyna 24453 (ANSM, US); 7.5 km E of Puentes on dirt road, Peterson, Valdes-Reyna & Sosa-Morales 17852 (US);14 mi S of San Roberto along Hwy 57, McGregor et al. 472 (KAU, NY, TAES, US); 7 mi E of San Roberto along Hwy 60, McGregor et al.439 (KANU, TAES, US); 25 km E of San Roberto along Hwy 58, Hatch et al. 4566 (TAES); 12 mi N of Matehuala, 0.5 mi N of SLP state line, Gould 10654 (TAES, UC); Ca. 10 mi NW of Rancho Margareta headquarters, 75 mi NW of Sabinas, Gould 10705 (TAES); 5 mi S of Monterrey on road to Chipinque, Gould 6312 (MICH, TAES, UC); Between Old Mexico City Hwy and El Diento, Monterrey, Smith M558 (TEX); 11 mi NW of Linares, Johnston 4644A (TEX); Galeana, Rancho Aguililla, Hinton et al. 19639 (TEX); Galeana, bank of steam, Chase 7742 (GH, MICH, MO, NA, NY, US); Monterrey, Hitchcock 5517 (US); Rancho Aguililla, Hinton et al. 19556 (ENCB, TEX); Sierra Madre Oriental, El Salero, Peterson & Valdes-Reyna 15817 (US). Oaxaca: Alongditch between Tule and Oaxaca, Hitchcock 6186(BISH); Cerro La Torrecilla al W de El Enebro, Mpio. Concepción Buenavista, Tenorio & Romero 9374 (TEX); road leading from Hwy 190 to nativity site of Benito Juárez, Sierra de San Felipe, Soderstrom 436 (US); Cerro La Torrecilla al W de El Enebro, Mpio. Concepión Buenavista, Tenorio & Romero 9374 (ANSM); 13 km E of Mitla on MEX Hwy 179 towards Ayutla, Peterson & Annable 9875 (BISH, K, US); 9 mi S of San Cualimojoyas, Peterson & Saarela 22334 (SERO, US). Puebla: Camino de Cholula a Acatepec, después de una curva doble en la salida sur de Cholula, Vibrans 3163 (MEXU); Rio de San Francisco, Puebla, Purpus 4079 (UC); 5 km al E de Santa Catarina Tehuixtla, ca. 5 km al E de Tepoztlán, Medrano et al. F–1372 (MO); Tehuacan, Purpus 1465 (RM); vicinity of San Luis Tultitlanapa, Puebla, near Oaxaca, Purpus 3591 (MO, NY, UC, US); 9 km NW of San Lorenzo on the Tehuacán-Tecamachalco Hwy (No. 150), Davidse & Davidse 9305 (MO, NY); 5 km carretera Tehuacán-Teotitlán, Mpio. Tehuacán, Morales 16 (MO); Afueras de Tehuacán, Puebla, por la carretera a Esperanza, González et al. F–312 (ANSM); Near Tehuacan, Pringle 9552 (GH, MO, TAES, US); Just over the Puebla-Veracruz boundary on Hwy 150, Gould 14904 (TAES); Mpio. Azumbilla 10 km al NE de Azumbilla, carretera a Esperanza, Tenorio 15209 (ARIZ, TEX); Oriental, Mpio. Oriental, Ventura 4148 (KANU, MICH); Vicinity of Puebla, Arsène 231 (CM, GH, MO, US). Querétaro: Querétaro, Escamilla 3 (MEXU); Mpio. Cadereyta, along MEX 120, ca. 3.5 km S of Vizarrón, Steinmann 3682 et al. (ARIZ); Ladera oriental de Cerro de la Tembladera, 6 km al N de Peña Blanca, Mpio. Peñamiller, Zamudio 3455 (ANSM); 8 mi SE of Queretaro, Gould 10235 (TAES, UC); 5 mi N of Queretaro, Gould 11597 (TAES, TEX); 11 mi N of Ixmiquilpan, Gould 9566 (TEX); 1 km al S de Vizarrón, Mpio. Cadereyta de Montes, Zamudio 3367 (MO, TEX); Near San Juan del Rio, Rose et al. 9587 (US); 4 km E of San Javier, Peterson, Romaschenko & Zamudio Ruiz 24742 (CIIDIR, IEB, US). San Luis Potosí: El Huizache, 11 mi E along Hwy 80, McGregor 748 et al. (KANU);Matehuala, 9 mi S along Hwy 57, McGregor 527 et al. (KANU);San Luis Potosi, 10 mi E along Hwy 86 to Rio Verde, McGregor 660 et al. (KANU); San Luis Potosi, 12 mi N along Hwy 57, McGregor 551 et al. (KANU); 32.2 km SW of San Luis Potosi on Hwy 70 to Aguacalientes, Peterson & Annable 6199(BISH, US); 30 km SW of San Luis Potosi on Hwy 70 to Aguacalientes, Peterson & Annable 6212(US); Sierra Madre Oriental, 2.5 mi E of Hwy 57 on road towards Guadalcazar, Peterson & Knowles 13389(BISH, K, US); Estación San Bartalo, Mpio de Rio Verde, Bravo 16 (MEXU); On border N of Saldana, Beetle et al. M–1768 (UC); San Francisco, 20 km al NE de Rioverde, Rzedowski 5150 (US); 12 km al W de la Est. Berrendo, Mpio. de Charcas, Rzedowski 6570 (US); 10 km al S de Cárdenas, Rzedowski 4598 (US); just S of Nuevo Leon/San Luis Potosi border on Hwy 57, Peterson, Saarela & Romaschenko 23233 (CIIDIR, US); 5.7 mi E of Wadley, Peterson, Saarela & Romaschenko 23275 (CIIDIR, US); 4.1 mi E of Charco Blanco on road towards Guadalcázar, Peterson, Saarela & Romaschenko 23338 (CIIDIR, US); 10 mi W of Guadalcázar, Peterson & Romaschenko 24665 (CIIDIR, US); 6.1 mi N of Lazaro Cardenas, Peterson & Romaschenko 24601 (CIIDIR, US); 7.1 mi W of Santo Domingo, Peterson & Romaschenko 24905 (CIIDIR, US); Peñasco, Rzedowski 3424 (US); Cerro al W de Villa Hidalgo, Rzedowski 3776 (US); Ca. 3 km E of Laguna Seca on NW slope of the Sierra de Alvarez, Sohns 1089 (P, TAES, US); On boarder N of Saldana, Beetle et al. M–1768 (FLAS); Valley of the Rio Verde and in the Sierra de Cuates, Sohns 1262 (P, TAES, US); 2 km S of Venados, on road to Moctezuma, Chiang et al. 8219B (LL, MEXU, NY); Mpio. Matehuala, Ejido Cerrito Blanco, 10 km al E de Matehuala, Lemus 123 (TAES); 10.4 mi E of El Huizache along Hwy 80 at K182, Henrickson 6533a (ARIZ, LL); 6.5 road mi S of Arista, Henrickson 6431 (LL); Carretera de S. L. Potosí a la Presa de San José, Gómez–Lorence 57 (ANSM); 3 km S of Jct with Hwy to Villa de Reyes, Reeves 6325 (LL); Cerro al W de Villa Hidalgo, Rzedowski 3776 (GH, LL); Hills above San Luis Potosí ca. 5 mi SW of the city, Reeder et al. 1370 (RSA); 18 km al E de San Luis Potosí, sobre la carretera a Rioverde, Rzedowski 11214 (MICH); Cerro Ventoso, 5 km al NE de Pachuca, sobre la carretera a Real del Monte, Rzedowski 19968 (MICH); 1 km al S de Cerro Godo, Mpio. Zaragoza, Rzedowski 11253 (MICH, TAES); Charcas, Whiting 486 (MEXU, MICH, US); Mpio. Villa de Arriaga, 2 km al W de La Placa, Gómez 878 (ENCB); Entroque a Derramaderos, Mpio. Ahualulco, Gómez s.n. (ENCB); Cárdenas, Hitchcock 5739 (US); Charcas, Whiting 957 (ARIZ, MICH, US); Charcas, Whiting 779 (MICH, US); Guascama, Purpus 5434 (GH, MO, NY, UC, US); Mpio. Villa de Arriaga, Rancho “El Palmar”, Potrero “Tortugas”, al SO del Edo., Rivas & González 197 (TAES); 41 mi S of San Luís Postosí, Gould 11579 (TAES, UC); 4.1 mi E of Hwy 57, 1.2 mi W of El Aguije, Hatch et al. 4878 (TAES); 5 mi SE of San Luis Potosí, Gould 11565 (TAES, UC, US); 24.1 km NE of San Luis Potosí on MEX 57 towards Matehuala, Peterson & Annable 11125(BISH, K, MO, NY, US). Sonora:51.6 km E of Agua Prieta on Hwy 2 towards Janos, Peterson & King 8138(US); Sierra la Mariquita, 9.4 im (air) NNW of Cananea, Reina-G. 2010-852 et al. (ARIZ); Sierra San Luis, Arroyo Las Cabañas, Rancho Los Pinitos, 61.3 km (air) ESE of Agua Prieta, Reigna-G. 2009-1221 et al. (ARIZ); Isla Tiburon, E side of the island, 1 km inland Zozni Quimpla, at base of N side of Punta San Miguel, Bilder 06-371 et al. (ARIZ); Mpio. Yecora, 19 km, al W de Yecora, Carr. a Cd. Obregón, Tenorio et al. 4561 (MEXU); La Vega Azul, SW of Colonia Morelos, Vera Santos 2171 (GH, TAES, TEX); Isolated hill NE of Sierra Anibacacachi, Rancho La Calera, ca. 10 km (by air) SW of Agua Prieta, Reinga G. 2003-1258 & Van Devender (ARIZ); Pinacate Region, arroyo half way between Tinaja de los Papagos and small “jungle-like” playa, ca 15 km S of MacDougal Crater, Eacurra s.n. (ARIZ 270781); Along Mex. Hwy 2, 16.6 km E of Agua Prieta, Reeder & Felger 8080 (ARIZ, MEXU, TEX); Valle de Teras, near La Angostura, White 3547 (GH, MICH, US); Horconcitos, Arroyo del Salto, White 3750 (GH, MICH); Cañon del Agua Amarga, White 3606 (GH, MEXU, MICH, NA); El Cañón de la Mescalera, Sierra de la Cabellera, Vera Santos 2116 (MICH); Agua Zarca, S of Colonia Morelos, Vera Santos 2010 (MICH, NY); El Puerto del Molino Quemado, E of Colonia Morelos, Vera Santos 2043 (MICH); La Vega Azul, SW of Colonia Morelos, Vera Santos 2171 (MICH, US); Guaymas, Palmer 273 (US); Nogales to Cocospora ranch, Griffiths 6804 (US); 6 km W of Los Vidrios on Mex. Hwy 2, Felger et al. 92–966 (MEXU). Tamaulipas: Mpio. Tula, 30 km al SW de Tula, cerca de límite de estados (SLP y Tamps.), González–Medrano 4432 (MEXU); El Canelo Ranch, 24 mi N of San Fernando on the Matamoros Hwy, Johnston 4879 (MICH, TEX); 5 km from San Fernando on the Victoria Hwy, Martínez and Borja F–2406 (TEX); 35 km from Victoria on the road to Casas and Soto la Marina, Martínez & Borga F–2347 (MEXU, TEX, US). Zacatecas:45.5 km NW of Fresnillo on MEX Hwy 45 to Durango, Peterson 9661(BISH, K, MO, US); 5 mi N of Cardona, Johnston 7371 (GH, US); 22 km N of Fresnillo rumbo a Durango, Beetle M-7483 & Yatskievych (ARIZ); 20 km N of Fresnillo, rumbo a Durango, Beetle M–7474 (ANSM); Intersection of Mexico 54 and the Tropic of Cancer, Brunken & Perino 476 (MO, TAES); 40 km N of Fresnillo rumbo a Durango, Beetle M–7504 (ENCB, TAES); 76 mi NE of Zacatecas (road junction with Hwy 45), Gould 12347 (TAES, US); 15 (air) mi NE of Estacion Camacho on NW slopes of Pico de Teyra, Henrickson 13424 (LL); 81 mi SE of Durango on Hsy 45, Pratt 614 (TEX); 71 mi SE of Durango on Hsy 45, Pratt 606 (TEX); 15 km al N de Fresnillo, Diaz Luna 988 (RSA); Near Conception del Oro, Palmer 268 (GH, NY, UC, US); Mpio. Concepión del Oro, Ej. Neria de Guadalupe, Del Rio s.n. (ENCB); Along Hwy 54, 0.4 road mi S of Hwy 60 turnoff towards Fresnillo, at junction of dirt road leading (unmarked) to Ranchito San Ramon, Snow 6673 (MO, MEXU); Near Sombrerete, Peterson, Saarela, Flores Villegas 21282 (CIIDIR, US); 20 km N of Fresnillo rumbo a Durango, Beetle M–7466 (MO); 10 mi E of Zacatecas at Guadalupe, Beetle et al. M–1793 (UC); 0.3 mi N of Chiripe, Peterson & Romaschenko 24845 (CIIDIR, US); Nombre de Dios, 48 mi SE, Marsh 1898 (KANU). Yucatán. About 17 mi SW of Mendoza, Reeder & Reeder 2017 (ARIZ). Peru. Ancash, Corongo, Peterson & Soreng 21791 (US, USM); Urubamba: Alturas de Tarapata, Vargas 13770 (US); Laderas de Muyock, Vargas 14109 (US). Puerto Rico. Río Piedras, Garcia M. 352(UPR). United States of America.Arizona: Apache Co., Big Canyon, Ft. Apache, Goodding & Schroeder 387–41 (RSA). Cochise Co., Ca. 14.3 mi NE of Douglas, along US Hwy 80, ca. 50 m N of mile marker 384, Snow 5857 (MO); 17 mi E of Douglas, Gould & Haskell 4518 (ARIZ);10 mi E of Benson, Anderson & Collins 478 (TAES); Tombstone, along First St., between Toughnut St, and Old Charleston Rd, Reeder & Reeder 8674 (ARIZ); 16 mi SW of Tombstone, Gould 7970 (TAES, UC); Southwestern Research Station, Chiricahua Mts, “Chiricahua Veg. Team” 59-798 (ARIZ); 1 mi W of Chiricahua Nat. Monument, 45 mi SE of Tucson, Deaver 6613 (TAES); AVA Ranch, Portal, Barr 67-319 (ARIZ); 9 mi S of Fry, Gould 2411 et al. (ARIZ); Pearce, Griffiths 1943 (ARIZ); Bisbee, Deaver 6625 (TAES); Entrance to Cave Ck. Canyon, Barkley 14A671 (RSA) and Barkley 14A678 (TEX, US); 6 mi S of Ft. Huachuca, Benson 11497 (POM); Ft. Huachuca, Wilcox 384 (POM); Ft. Huachuca, Gooding 853 (ARIZ); 9 mi S of Fry, Gould et al. 2411 (POM, UC); Triangle Tree Rd.-Dragoon Turnoff from I–10, ca. 13 mi E of Benson, Davidson 6430 (POM); Dragoon Summit, Tornber s.n. (ARIZ 139150); 10 mi E of Benson, Collins 478 (ARIZ); Dragoon Mts, ca. 1 mi SE of Sheepshead Pass, Yatskievych 85-293 & Windam (ARIZ); Dragoon Mts, Griffiths 1859 (ARIZ); Cave Creek, Chiricahua Mtns, Shreve 6351 (ARIZ); Arizona Hwy 181, 2.5 mi W of Chiricahua National Monument Headquarters, Strandberg 344 (ARIZ); Ditto, 4 mi W of Chiricahua N.M. Hqrts, Strandberg 348 (ARIZ); Hwy 181, 1 mi W of Chiricahua N.M., Wilcox 6613 (ARIZ); El Coronado Ranch, Turkey Ck, Chiricahua Mts, Miller G-470 (ARIZ); Near Marble Canyon (1 mi SE of Marble Camp), Dos Cabezas Mtns, Kaier s.n. (ARIZ 20544); San Pedro Riv., ca. 1 km E of Benson, Baker 10173 (ARIZ, RSA); San Pedro Riparian National Conservation Area, “Palominas-3” site, ca. 1 mi S of Palominas Rod, Makings 513 (ARIZ); Tinker Canyon 11th Brigade Signal Corps site, 0.3 km NW of mouth of Tinker Canyon, 3 km E of the mouth of Garden Canyon, Schulz & Krohn 2496 (ARIZ); West Gate 11th Brigade Singal Corps site, 0.5 km NW of Kino Springs, Kronh & Shulz 2106 (ARIZ); Ditto, 1.0 km N of the mouth of Huachuca Canyon, Morrison & Popolizio 1194 (ARIZ); Ditto, immediately SW of Buffalo Soldier Road, 2.4 km S of Main Gate, 2.5 km NE of Stone Ridge, Schulz & Krohn 2667 (ARIZ); Along AZ-90 ca. 17 km S of jct with I-10, Reeder & Reeder 9750 (ARIZ); Carr Peak, Benson 10487 (RSA); AVA Ranch, Portal, Barr 67–319 (NCU); Gate 7 at Saddle above Scotia Canyon and Boundary of Fort Huachuca Military Reservation, ca 2.5 mi NE of Sunnyside, Peterson & Annable 5476(US); Chiricahua Mts., 10 mi S of Rucker Canyon Rd. on Tex Canyon Rd. and 6 mi NE of HWY 80, Peterson & Annable 5524 (BISH, US); Rucker Canyon, Gould & Haskell 4556 (ARIZ); Rucker Canyon, Chiricahua Mts, Jones & Thornber s.n. (ARIZ 139157); Ramsey Canyon Nature Preserve, Miller Peak Quad, T23S, R20E, SE1/4 of SW1/4 of Sect. 9, Adams 203-83 (ARIZ). Coconino Co., Prescott, Purchase 416 (MICH) and Purchase 3383 (ARIZ); Prescott, Benham 846 (ARIZ); Highway above Jerome, Goodding 72-46 (ARIZ); Sycamore Canyon Wilderness Area, Pinkava et al. 5902 (NCU); Ditto, 150 m N of Yavapai County line, Maker 10128 (ARIZ). Gila Co. 3 Bar Study Area, Tonto National Forest, Pase 1173 (ARIZ); Lower Parker Ck. Canyon, near south end of Natural Drainages Experimental Area A., Sierra Ancha Mts., Gould & Hudson 3837 (ARIZ, US); Parker Creek Exp. Stn., Hendricks 2052 (RM); Branch of Pinto (Riv.), Read 16 (RM); Sierra Ancha Wilderness Area, along FSR 203 ca. 6.2 mi S of Board Tree Saddle (jct with Hwy 288), Imdorf 1017 & Rebman (ARIZ); Sierra Ancha Wilderness Area, just N of Bull Canyon trailhead (trail 128) at end of FSR 203A, Imdorf 1045 & Dow (ARIZ); Pinal Ranger Stn., Sec. 22, T1S, R15E, Kirby P-2 (RM). Graham Co. Frye Mesa, Graham Mts., Kessler 500 (NY, TAES); 16 mi S of Safford near the highway, Gould & Haskell 3994 (ARIZ); BLM Wilderness, Black Rock, Buegge 622 (ARIZ); Swift Trail Rd, Maguire et al. 12099 (ARIZ, RM); Frye Mesa, Graham Mts, Kessler 500 (ARIZ); Sec. 10, T9S, R23 E, Christensen MG-32 (RM). Greenlee Co. Benton Creek near Baseline R.S., Sizer 30 (RM); 5 km NE of Greenlee-Graham Co. line along US-191, Reeder & Reeder 9176 (ARIZ) and 9621 (ARIZ); Along US-191; Big Lue Range, near Hwy 78 ca. 7 mi from the state line, Gould & Haskell 4109 (ARIZ, UC); Honeymoon, Eagle Creek, Pase 2270 (RM). La Paz Co., Eagletail Mts., side of Indian Spring Canyon, Newton 10 (ASU). Maricopa Co. US Hwy 60, 2.6 mi E of Queen Ck. Tunnel, Pinkava et al. L18913 (ASU, FLAS); Hassayampa River Preserve, ca 2 mi SE of Wikenburg on Hwy 89, Woldon 607 & Richter (ARIZ); Tonto National Forst, ca. 0.1 mi S of Seven Springs campground along road, Doan 931 (ARIZ); Sonoran Desert National Monument, Sand Tank Mts, north-facing slope ca 50 m eastward from summit of small peak, 730 m (air) NW of Bender Spring, 250 m northward from end of Bender Spring road, 1.05 km (air) NNW of Squaw Tit Peak, Felger 01-384 et al (ARIZ); Agua Fria River bottom near Cold Water, Harrison 1813 (ARIZ). Pima Co., Molina Canyon, Santa Catalinea Mts, R.S. Felger 668 (ARIZ); Stone Cabin Canyon above McClearys Camp, Hill 356 (RM); Santa Rita Range Reserve, Southwest Pasture no. 6, Culley 54 (RM); ditto, Pasture 2b, Streitz S-56 (RM); Canille Ranger Station, Rodgers 2 (RM); Near Florida Station in Canyon, Magee 385 (RM); Oracle Ranger Stn., T10S, R15E, Read 37 (RM); Rincon Ranger Stn. pasture, Galer 2 (RM); Santa Catalina Mts, 3.6 mi S of General Hitchcock Campground on Mt. Lemmon Hwy, Peterson & Annable 5656 (US); Santa Rita Mts, 2.1 mi W of Hwy 83 on Forest Service road 92 up Gardener Canyon, NW of Sonoita, Peterson & Annable 5684 (US); Santa Rita Mts. 0.4 mi W of Hwy 83 on FS 231 towards Rosemont Springs, Peterson & Annable 12235(K, US); Santa Rita Mts, Thornber Griffiths 77 (ARIZ); Santa Rita Mt, J.W. Toumey s.n. (17 Jul 1894) (ARIZ); Tanque Verde Ck., 7 mi below Italian Ranch, 18 mi E of Tucscon, Tanque Verde Mts., Parker 7164 (ARIZ, MICH); Southern Comobabi Mts., near Comobabi, Gould & Haskell 3206 (ARIZ, GH, NY, UC); SW slopes of Santa Catalina Mts., near Mt. Lemmon Rd., Gould 3451 (ARIZ, TAES); Nolina Basin Recreation Area, Sta Catalina Mts., Parker 7087 (ARIZ, RSA); Crest of Gates Pass, Tucson Mountain Park, Van Devender 88-798 (ARIZ); Tucson Mountains, Oeste Wash; Van Devender et al. 88-595 (ARIZ); Tucson Mountains, near radio towers on crest, nw of Trails End Canyon, Van Devender & Van Devender 88-698 (ARIZ); Cienega Creek Natural Preserve, next to train tracks at Three Bridges, near parking area below Marsh Station Road, Mauz 24-097 (ARIZ); Tucson, in roadside ditch 4 mi N of Tucson on Campbell Ave, Gould 2523 (ARIZ); Tucson, Sta Cruz Riv., Benson 8935 (POM); 61 mi SSE of Tucson, the Research Ranch, Elias 8976 (NY, RSA); 5 mi W of State Hwy 83 on Greaterville-Madera Canyon Rd, McLaughlin 61 (ARIZ); Sabino Canyon, Sta. Catalina Riv., Benson 9829 (RSA); Florida Canyon, Santa Rita Range, Benson 8974 (POM); Santa Rita Experimental Range, Galt s.n. (ARIZ 168258); Ca. 4.0 mi W of SR 83 along Greatville-Box Canyon Rd, Gutierrez 1756 (ARIZ); Baboquivari Cañon, Peebles et al. 575 (ARIZ, US); Baboquivari Canyon, Peebles et al. 346 (ARIZ) & 575 (ARIZ, US); Baboquivari Mts, Goodding 844 (ARIZ); Baboquivari Canyon, W side of Baboquivari Mountains, 2 mi from end of road that leads in from highway, Leader & Leader 375 (ARIZ); Thomas Canyon, E side of Baboquivari Mts., Toolin 398 (ARIZ); Mendoza Canyon, Coyote Mountains, Kurt & Haskell 45 (ARIZ); Ajo Mountains, main canyon N of Alamo Canyon, Gould & Darrow 4690 (ARIZ); Molina basin, Catalina Mts., King 229 (NCU); Rincon Peak, 20 km SE of the edge of Tucson, 2.5 km WSW of Papago Spring, Baker 16350 (ARIZ); Ragged Top Peak, ca. 4 mi N of Silver Bell, Wiens et al. 89-RT-43-02 (ARIZ); Arivaca Cienega, Buenos Aires Natioanl Wildlife Refuge, McLauglin 6033 (ARIZ); Brown Wash, McLaughlin 4914 (ARIZ). Pinal Co., Pinal Mts. in pass S of Globe, Shreve 7459 (MICH); Oracle Road, Santa Catalina Mountains, Niering & Whittaker 388 (ARIZ); Above Oracle, Peebles 2559 et al. (ARIZ); Oracle, Thornber s.n. (ARIZ 139146); Ditto, Thornber s.n. (ARIZ 139160); West end of Holy Joe Peak, Galiuro Mtns, Darrow s.n. (ARIZ 14848); Peppersauce Canyon, Control Road, Santa Catalina Mtns foothills, Leader & Leader 332 (ARIZ); Sun Space Ranch, along arroyo WSW of production complex on north-facing slope, Burges 7139 (ARIZ); Pinal Mts, in pass south of Globe, Shreve 7459 (ARIZ). Santa Cruz Co., Sonoita Creek State Park Natural Area, saddle along ridgetop, McLauglin 8421 & Lewis (ARIZ); San Rafael State Park, along E boundray fence, McLauglin 8582 et al. (ARIZ); Ditto location, McLaughlin 8575 (ARIZ); Ditto location between Sharp and Heron Springs, McLaughlin 8707 & Lewis (ARIZ); The Research Ranch, drainage leading to East Tank, McLauglin 10054 (ARIZ); Ruby Road 2 mi W of US Hwy 89, Barr 60-279 (ARIZ); San Rafael State Natural Area, San Rafael Valley to immediate W of Santa Cruz Riv., 0.5 mi N of Mexico border, Reif 10478 (ARIZ, RM); W slope of Atascosa Mts., Sycamore Canyon, Franklin 5356 (NY, RM); Nogales, Peebles & Harrison 4674 (ARIZ, US); Patagonia Nature Conservancy Wildlife Sanctuary, Correll & Correll39284 (LL); Patagonia-Sonoita Creek Sanctuary, Along Sonoita Ck, less than 1 mi S of Patagonia, Rogers 30 & Fassuliotis (ARIZ); Cornado National Forest, ca. 4.0 air mi N of Mexico border, Jct of USFS roads 39 and 115, Snow 5865 (MO); 9.3 mi NE of Patagonia along AZ Hwy 82, Snow 5867 (MO); Madera Canyon, Parker 7077 (ARIZ, RSA); Nogales, Thornber s.n. (ARIZ 139153) Nogales, Jones 22760 (POM); Appleton Whitehall Research Ranch, near Elgin, ca. 61 mi SSE of Tucson, Lyle Canyon, near S boundary of Ranch, Thomlinson et al. 747 (NY, RSA); Ditto, northeast of headquarters, McLaughlin 7334 & Plemons-Rodriguez (ARIZ); Ca. 5 mi N of Elgin, Reeder & Reeder 6955 (ARIZ); Monkey Springs Area, Goodding 150–62 (ARIZ, NCU); Patagonia, Hitchcock 3651 (US); Along the road from Canelo Pass to Patagonia, ca 3 mi SW of the Pass, in the north end of the San Rafael Valley, Toolin 1133 & Reichenbacher (ARIZ); 5 km E of Washington Camp, Reeder & Reeder 7418 (ARIZ); 3.3 mi W of HWY 289 and Pena Blanca on Forest Service Rd. 39 towards Sycamore Canyon, Peterson & Annable 5320(BISH, K, US); O’Donel Canyon, N of Knipe Cienega, Yatskievych 80-514 (ARIZ); Patagonia Mts, 1.7 mi SE of Patagonia on Harshaw Ck., Peterson & Annable 5387 (US); Ophir Gulch, 4 mi N and 2 mi W of Sonoita, Ptramontano T 91 (ARIZ); Sycamore Canyon, Pajarito Mountains, Van Devender s.n. (ARIZ 201184); Sycamore Canyon, Pajarito Mountains, on east-facing slope immediately W of Hank & Yank ruins, Toolin 2128 & Buhrow (ARIZ). Yavapai Co., 2 mi N of Mingua Mt. Pass, Prescott to Jerome, Benson 14223 (POM); Tributary of Little Shipp Wash, ca. 9 mi SE of Bagdad, ca. 3.5 mi W of Santa Maria River, 1.5 mi N of AZ 96, Van Devender 91-798 et al. (ARIZ); Santa Maria River, canyon on W side of crossing, Darrow & Gould 3656 (ARIZ). Yuma Co., Ten Ewe Canyon, Kofa Mts, Monson 5 (ARIZ); Wire Corral, 13 Mile Rock Allotment, Julander 113 (RM). Florida. Collier Co., Collier City (Caxambas) on Marco Island, Deam 65392 (US); Marco Island, beach and hammock N of Caxambas Pass off US 92, Lakela 29242 (GH); Goodland, Godfrey 65552 (FSU); Caxambos, Garber 33 (GA, GH, MO, NY, US); In sand dunes, Caxambos, Moldenke 999 (MO, NY, US). Lee Co., Pointybel, Sanibel Island, Brumbach 5809 (FLAS, GH); Darling Sanctuary, Sanibel Island, Brumbach 7294 (NA, FLAS, US); Vicinity of Fort Myers, Standley 18946 (US); Sanibel Island, Cooley 2659(US); Western Sanibel Island, Wulfert, Brumbach 8666 (GA, NCU, NY, US); Lower Camtiva Island, Brumbach 9280 (GA, GH, NCU, US); Sanibel Island, waste land near entrance to Sawgrass Rd., Cooley 2309 (FLAS, GA, NCU). Monroe Co., Key Largo, Phillips 588 (ARIZ); North Key Largo ca 3 mi from left turn of US Hwy 1, Cooely et al. 9257 (FSU); Vaca Keys, Small & Carter 2860 (FLAS, GH, US); Everglades N.P., Northwest Cape Sable, Avery & Russell 2138 (FLAS); Big Pine Key, Swallen 10670 (US); Big Pine Key, Small et al. 3572(US); Big Pine Key, Killip 40685 (US); Big Pine Key, Killip 41238 (US, VPI); Big Pine Key, Killip 41715 (GA, GH, US); Big Pine Key, Radford 46083 (NCU); Big Pine Key, Martin 1323 (NA, NY, UC); Big Pine Key, Killip 32092 (UC, US); Sugarloaf Key, Sargent 6693 (GA); Key Largo, Hitchcock 675 (LL, GH, MO, RM, UC, US); N end of Key Largo, Kral 51801 (MO, TEX); West Summerland Key, Godfrey 74051 (FSU, NCU); Key Largo, Atwater GS–182 (NCU). Sarasota Co., Longboat Key, south end, beach hammock overlooking Gulf of Mexico, Long & Lakela 27563 (FLAS, GA). Hawaii. Maui Co., Molokai: Molokai Project – Transect 9, Makakupa’ia Ridge, Char et al. 82.046(HAW); Kawela, east of Road TRA, A-1300 plot, Jacoby s.n.(BISH). Kansas. Douglas Co., Lawrence, Booth s.n. (KANU, MONT 38814). Missouri. Crawford Co., In test plot, Pickle 150 (UMO). St. Clair Co., Tony s.n. (UMO 147246). New Mexico.Chaves Co.: Along U.S. Hwy 82, ca. 20 mi E of Mayhill, Henderson 69–314 (FSU, MO, NCU). Dona Ana Co., Foothills of Organ Mts., Barneby 2464 (NY); South end of Bishop’s Cap, Van Devender s.n. et al. (ARIZ 192727); On US Hwy 70–82 2.7 mi E of San Augustin Pass, Spellenberg & Spellenberg 3684 (NY); Bishop Cap, 2 air km NNW of the top of Bishop Cap, Worthington 17281 (NY); Mesa W of the Organ Mountains, Wooton 3198 & Standley (ARIZ); Aguirre Springs, Hwy 70, 15 mi from NMSU in Las Cruces, Adeola 37 (TAES); 4 mi from Organ Mt., on the way to White Sands Nat. Museum, Dashe 3 (TAES); 13 mi E of Las Cruces, ca. 1/4 mi SE of Hayner Ranch, Dunn 8576 (RSA); Canyon in S end of Organ Mts., Fosberg 53671 (POM); Organ Mts., Wooton 418 (GH, NMC, NY, P, POM, RM, UC, US); Dripping Springs in Organ Mts., Archer 499 (MICH); Dona Ana Mts., S end of College Ranch, 15 mi N of Las Cruces, Hatch 2336 (TAES); Filmore Canyon, Organ Mts., Hitchcock 3798 (MICH); Jornada Range Reserve, below Goldenburg Spring, Copple 328 (RM). Eddy Co.: Guadelupe Mts near Queen, Hitchcock 676 (RM); Jornada R.R., 1.5 mil W of Ropes House, Pasture 11, Schoeller & Hough 417 (RM); Carson Seep Ranger Station Pasture, Chapline 452 (RM). Grant Co.: Mangas Cañon, 16 mi WNW of Silver City, Barkley 14653 (TEX, US); 18 mi NW of Silver City, Metcalfe 641 (ARIZ, GH, NMC, NY, RM, US). Guadalupe Co., Vicinity of Santa Rosa, Arsène & Benedict16973 (P). Hidalgo Co.: Little Hatchet Mts., Granite Pass, Worthington 12604 (NY); Apache Hills, slopes about the Apache Mine, Worthington 13498 (NY); Animas Mts., ca. 7 air mi SE of Animas, Worthington 14788 (NY); Guadalupe Canyon, ca. 1 mi E of AZ state line, Allred 4282 (TAES); Box Canyon near Lynch Camp, Turner & Felger 98-133 (ARIZ); Saddle in Spring Canyon Range, Pigott 7a (RM). Lincoln Co., N side of Capitan Mts., New Mexico route 48, 12 airline miles E of Encinoso, Holmgren & Holmgren 7416 (KANU, NY, NMC, RSA); Corona, Hershey s.n. (GREE). Luna Co.: Tres Hermanas Mts., canyon on NE side of South Peak, Worthington 19818 (RSA). Otero Co., 9 mi S of Alamogordo on W face of Sacramento Mts, S slopes of mouth of Dog Canyon, Spellenberg & Spellenberg 4296 (NY); McKittrick Canyon, Patterson 610 (TEX); N McKittrick Canyon, Patterson 537 (LL); Road to Rinconada, Humphrey 59 (ARIZ). San Miguel Co., Conchas Canyon below Crystal Pasture, 8.5 mi due W of Trujillo, Hill & Levandoski 12214 (TAES); Cuevas Canyon Rd. cut at Conchas Canyon below Crystal Pature, 8.5 mi due W of Trujillo, Hill and Levandoski 12214 (GH, NY). Sierra Co.: 6 mi NW of Hillsboro off Hwy 90, Milestone 37 (TAES); W slope of the Caballo Mts., 6.7 mi by winding road E of Caballo Dam on the Rio Grande, Spellenberg 3920 (NMC, NY); San Andres Mtns, White Sands Missile Range, Rhodes Canyon, 0.1 mi SW of Bearden Canyon Rd, just above Granite Gap, Van Devender & Toolin 1903 (ARIZ); Ridge N of Trujillo Canyon, Chapline 377 (RM); Gottom [of] south Percha Creek, Chapline 623 (RM). Oklahoma. Cimarron Co., Side of Mesa de Maya (Black Mesa), 3.5 mi N of Kenton, Rogers 6411 (MICH, TEX, US). Murray Co., On rocky bank of mt. stream, Arbuckle Mts., Hopkins 1109 (GH, MO, US); Arbuckle Mts., Cowpen Canyon, Hopkins et al. 715 (GH, RM); Ca. 0.5 mi W of Honey Ck., Arbuckle Mts., Robbins 3175 (TAES, UC); Igneous canyon wall along creek 8 mi W and 3 S of Davis, Waterfall 6743 (MO, VPI). Texas. Aransas Co., Cape Carlos, Tharp 9125 (TEX); Goose Island State Park, Brown 53–252 (TEX); Near seashore, Metz 3318 (NY). Armstrong Co., Claude, 15 mi S, 6 mi SW, Stephens 57414 (KANU). Bandera Co., Lost Maples State Park, confluence of Sabinal Riv. and Can Ck., Smith 698 (TEX). Bastrop Co., Bastrop State Park, Lybarger 100 (CM). Bee Co., 7 mi E of Beeville, Gould 6057 (TAES, TEX, UC). Bell Co., 8 mi N of Belton, Wolff 1308 (TAES); Near Moffat, Wolff 2681 (BRIT, TAES); Prairie near Schwertner, Wolff 2551 (US). Bexar Co., Leon Springs, Clemens & Clemens 75 (MO, MONTU); 15 mi NW of San Antonio, Burr 292 (TEX); US Hwy 45, 4 mi N of Richland Springs, England & McCart 9135 (TEX); San Antonio, Silveus 865 (US). Blanco Co., Pedernales Falls State Park, 7 mi N on Ranch Rd. 3232 off of US 290, E of Johnson City, Hill 3744 (TAES); 1 mi S of Johnson City, Francis 77 (TAES). Brewster Co., 1.5 mi E of Alpine, Gould 8317 (BRIT); Paisano Pass, 12 mi W of Alpine, Shreve 8354 (ARIZ); 7.9 km E of Alpine on U.S. 90, Van Devender 2003-841 & Reina (ARIZ); Glass Mountains, Warnock 20872(US); Paradise Canyon, 4 mi W of Alpine, Warnock 20195 (BRIT) and 20196 (BRIT, KANU); Kokernot Lodge Road, Alpine, Warnock 20197 (BRIT); Leonard Mt., Warnock 20750 (BRIT); Rio Grande at Mariscal Canyon, Warnock 811 (BRIT); Big Bend N.P., Inner Basin, Chisos Mts., Kruckeberg 4737 (UC); 13 mi S of Marathon, Wood Hollow Mts., Wallmo 5341 (TAES); Chisos Basin, Big Bend N.P., Gould 9722 (TAES); 18 mi S of Marathon, Parks 30155 (TAES); Along Hwy 90, 5 mi W of Alpine, Morden 69 (TAES); Del Norte Pass, Berkman 46208 (TAES); N slope of Elephant Mesa, ca 35 mi S of Alpine, Johnston 6435 (LL, NY); Big Bend N.P., Upper Green Gulch, Chisos Mts., Warnock & Tharp 6839 (BRIT, LL); Hwy between Alpine and Fort Davis, Teague T31 (BRIT, LL); Boot Canyon, Correll 13721 (BRIT); Big Bend N.P., in Oak Canyon below the “Window”, Correll & Correll30618 (LL); Chisos Mts., lower slopes of Basin, Lundell 13279 (LL); Kobernath Lodge Rd., Warnock 20197 (LL, POM); 9 mi NE of Alpine on O 6 Ranch, Warnock 21734 (TEX, US); N Side of Leonard Mt., Warnock 20750 (TEX, US); Paradise Canyon, Warnock 20195 (US); Glass Mts, Green Valley, Warnock W361 (US); Mt. slopes 12 mi W of Alpine, Shreve 8352 (MICH, US); Leonard Mt., Warnock 20750 (NY); Honeysuckle Canyon, Glass Mts., Warnock 21062 (TEX); Sul Ross College Campus, Alpine, Sperry T718 (UC); Chisos Mts, Upper Blue Ck., Mueller 7876 (GH, MICH, MO, NY, US); North Sunny Glen, Chisos Mts., Sperry T–491 (TAES, UC); Blue Ck. Canyon, Chisos Mts., Wolff 4476 (TAES). Briscoe Co., 9–12 mi N of Turkey on the Breaks of the Little Red Riv., Higgins 8263 (NY). Brooks Co., Near Huesos Camp, Encino Division, King Ranch, Morrow & Nord 30 (TAES). Brown Co., Brownwood, Palmer 29519 (MO, NY) and Palmer 26986 (GH). Burnet Co., 1 mi E of Bertram, Rogers & Webster 6467 (KANU, RM, TEX); Marble Falls, Biltmore 4155a (S, US). Caldwell Co., Edge of Wilcox farm site, Tharp & Tyson 52–540 (TEX); TX Hwy 80, 5.6 mi E of junction with FM 20, Breckenridge 549 (TEX). Cameron Co., Los Fresnas to Brownsville, Swallen 1427(US): 14 mi E of Brownsville on Hwy 14, Lonard 2825 (TAES); 10 mi W of Port Isabel on Hwy 100, Lonard 4988a (TAES); Laguna Atascosa Nat. Wildlife Ref., 2 mi from North Point, Fleetwood 7042 (TEX); Laguna Atascosa NWR., Unit 1, Fleetwood 7039 (TEX); Levee of Resaca de la Gringa, Johnston 542187 (TEX); Levee of Resaca de la Gringa in Laguna Atascosa NWR, Johnston 542176 (TEX); Laguna Atascosa NWR, along gunnery range, Fleetwood 3322 (TEX); Laguna Atascosa NWR, Fleetwood 3736 (TEX); Laguna Atascosa NWR, Unit 2, near impoundment 1, Fleetwood 8131 (TEX); near Brownsville, Point Isabel Rd., Runyon 132 (TEX); 8.2 mi from Boca Chica, NE of Brownsville, Lundell and Lundell14712 (GA, LL, TEX); New Barreda, on the R.R. right of way, Runyon 4073 (BRIT, TEX); Los Fresnos, Swallen 1440 (US). Comal Co., Near Canyon Lake Dam, Davies 97 (TAES); Payne’s Ranch on Smithson Valley Rd., 2 mi off of Hwy 46 W of New Braunfels, Fey 82 (TAES); 3 mi W of Hwy 281 N on Hwy 46, Edwards Plateau, Coltman 7 (TAES). Concho Co., 2 mi E of Eden, Gould 9528 (TAES). Crockett Co., Ca. 3 mi W of Ozona, along Texas FM 2398, Snow 5891 (MO). Culberson Co., 4 mi S of New Mexico State line on Hwy 62, Whitehouse 16941 (BRIT); About 4 mi NE of Pine Springs, Waterfall 5755 (BRIT); 6 mi NE of Pine Springs, Waterfall 5748 (GH, NY); Guadalupe Mts National Park, mouth of Pine Spring Canyon ca. 0.1 km NE of campground, Burgess 2321 (ARIZ); 7 mi N of Pine Springs, Davis et al. 243 (TAES); 9K Ranch, vicinity of Gray Tank on Willow Draw, 4.0 mi S, 6.5 mi W 9K headquarters, Burgess 653 (TAES); 9K Ranch, 1.8 mi E Ranch Rd. 1108, 2.8 mi N, 0.4 E 9K headquarters, Burgess 756 (TAES); Apache Mts., ca. 28 mi NW of Kent, Correll 31666 (LL); Guadalupe Mts., Pine Canyon, Standley 40611 (US); Creek, 7 mi NE of Pine Springs, Smith & Robertson 243 (US); Limestone hillsides approaching the Guadalupe Mts., ca. 4 mi NE of Pine Springs, Waterfall 5755 (GH, MO, NY); Pine Springs Canyon Trail near the Summit, Guadalupe Mts. N.P., Higgins 17468 (NY); Bear Canyon, Guadalupe Mts. N.P., Higgins 17428 (NY); Bear Canyon, Mount Lemmon Hwy, Santa Catalina Mts, Van Devender & Eiber s.n. (ARIZ); Upper Pine Springs, Guadalupe Mts, Turner & Warnock 85 (GH, LL). Duval Co., Texas Route 44, ca. 5 mi E of Duvall County Line near Freer, Seigler et al. 15326 (BRIT); 1 mi W of San Diego, Gould 10998 (TAES); 3 mi S of Freer, Tharp & Johnston 541817 (TEX); 9 mi SW of Freer on road no. 202, Tharp & Johnston 542023 (BRIT, RM, TEX). Edwards Co., Sonora Experimental Station, Gibbens 36 (TAES); Sonora Experiment Station, Nolen 34 (BRIT); Substation No. 14, Cory 52478 (BRIT, MICH, NY, RM, TAES); 14.5 mi SE of Rocksprings, Cory 35637 (TAES); T.A.E.S. Substation 14, ca. 25 mi S of Sonora, Gould 8391 (BRIT, TAES, UC); 1.1 mi N on the entrance raod along limestone ledges on Kickapoo State Park, Keeney 7424 (BRIT); Ca. 1/4 mi S of the dam on the W bank of Hackberry Ck. near Deadman’s Hollow, Smith & Butterwick 247 (LL); Substation No. 14, Cory 53478 (UC). El Paso Co., McKelligon Canyon, Franklin Mts., Shinners 9001 (BRIT); Franklin Mts., 0.9 air mi WNW jct. Trans–Mt. road & Gateway South, Worthington 9186 (BRIT, NY); El Paso, Jones 25a (US); McKelligan Canyon, Franklin Mts., Warnock 7373 (BRIT, LL, TEX); McKelligan Canyon, Franklin Mts., Correll 13834 (BRIT, LL, US); Mt. Franklin, Warnock 10354 (BRIT, LL); 5 mi W of El Paso, Warnock 7246 (TEX); El Paso, Jones 4210 (NY, POM, US); El Paso, Stearns 182 (US). Fannin Co., Ca. 1 mi NW of center of Honey Grove, Nee 44047 (BRIT, NY, TEX). Garza Co., 2.8 air mi SW of Post, Hutchins 939 (BRIT, LL). Gillespie Co., Kast Ranch, 18 mi N of Fredricksburg, Kast 9 (TAES). Goliad Co., McNamara Ranch, 2.8 mi SW of Goliad, Hill 6307 (TAES); 5.6 mi SW on US 59 from its jct. with FR’s 2957 & 2506 in Fannin, Jones & Jones 5931 (GA, NY); 3.5 mi N of Eckert (17.5 mi NE of Fredericksburg), Shinners 31085 (BRIT). Harris Co., Intersection of Underwood St. & Fairmont Parkway in Deer Park, Brown 8100 (TAES). Haskell Co., Ca. 3 mi W of Rochester, Grayum 46 (TAES). Hays Co., 6 mi W of San Marcos, Niemann 19–62 (TAES); Outside San Marcos, on Ranch Rd. 12 in the Devils Backbone, Caeser 19 (TAES); Blanco Riv., 7 mi NW from Kyle, Tharp 47 (LL, TEX); 7 mi W of San Marcos, Gould 6696 (BRIT, TAES, TEX); 5 mi S of Wimberly, Emery 806 (TEX); 2 mi SE of Wimberly across road from entrance to Hidden Valley Ranch, Johnson 422 (TEX). Hidalgo Co., Railroad crossing, Sprague Street, Edinburg, Lonard 5010 (TAES); Donna, alley by Post Office, Fleetwood 8044 (TEX); 5 mi N of Edinburg, Runyon 1892 (LL); 10 mi W of Mission, Lundell & Lundell 9969 (LL); S. O. Johnson home south of Alamo, Clover 1481 (US); Santa Ana National Wildlife Regue, Fleetwood 7059 (TEX). Hood Co., 2 mi E of Tolar on US Hwy 377, Vest 6 (TAES); Top of Comanche Peak, near Granbury, Palmer 6529 (MO). Howard Co., Big Spring, Tracy 8219 (CM, GH, MO, TAES, TEX, US). Hudspeth Co., Ca 30 mi E of El Paso (Hwy 180), Warnock 13819 (LL, TEX); 5 mi W of Van Horn, Beach Mts., Warnock 13635 (LL, TEX). Humble Co., Jesse Jones County Park N off of Hwy 1960 W of Humble, Rolling s.n. (BRIT). Jeff Davis Co., 10 mi NW of Valentine on US Hwy 90, Spellenberg & Spellenberg 3690 (NY); Valentine, Goodding A-9747 (ARIZ); 12 mi NW of Ft. Davis, Gould 7657 (BRIT, MO, TAES); 5 mi NE of Juno, Warnock & McBryde 15066 (LL); Mitre Peak Girl Scout Camp at Fern Canyon, Keough 58 (TEX); Limpia Canyon, 10 mi N of Fort Davis, Innes & Moon 1107 (TEX, US); Frazier Canyon, Davis Mts., Cory 12074 (GH); Davis Mts., 7 mi from cut–off to McDonald Observatory, Hwy 118, Lundell & Lundell 14227 (LL, US); Davis Mts., Little Aguja Canyon, Palmer 34549 (NY); 0.25 mi below McDonald Observatory turnoff along Hwy 118 NW of Ft. Davis, Hatch & Morden 4330 (MO, UC); Along Hwy 118 by rest stop; 1/4 mi E of turnoff to McDonald Observatory, Morden & Hatch 77 (TAES); 19.3 km NW of Ft. Davis on TX 118 & 61.3 km S of Kent, Peterson & Annable 10407 (BISH, K, US). Jim Wells Co., 5 mi S of Alice on Hwy 281 & 7 mi W of Ben Bolt, La Capita Research area, Coffey 806 (TAES); ditto, Coffey 820 (RM); NE side of SE arm of Naval Auxillary Landing Field, Orange Grove, Carr 11459 (TEX); 5.0 mi S of Alice & 7.0 mi W of Ben Bolt at the La Copita research area, Whitley 33 (US). Karnes Co., Cemetery Rd., Karnes City, Johnson 1051 (TAES); 2.3 mi NE of Karnes City, Johnson 991 (TAES, TEX). Kenedy Co., King Ranch, Norias Division, Lundell 14985 (LL, UC, US); Just N of Mifflin, Kenedy Ranch, Johnston 541575 (TEX); SW of El Toro Island, Tharp 48348 (TEX); El Toro Island, Tharp 49233 (NY, TEX); El Toro Island, Atnip 48–407 (TEX); 3 mi S of Norias, off Hwy 77, Lundell 14958 (ARIZ, LL, UC, US). Kendall Co., Boerne, Palmer 10860 (MO). Kerr Co., Kerr Wildlife Mgmt. Area, East Bobcat Pasture, Sanchez 4077 (BRIT); Mt. Tivy, Kerrville, Cory 50473 (BRIT, MICH, NY, US); 10 mi N of Hunt on Kerr Wildlife Mgmt. Area, Coffey 40 (TAES); 3 mi W of Kerrville, May 5517 (BRIT, TAES); 11 mi W of Hunt on FM 1340, Hyman 56 (TAES); Kerr Wildlife Mgmt. Area, Copeland 20 (TAES); Kerr Management Area, Hendley 33 (TAES). Kimball Co., 8 mi SE of Junction, Gould 9681 (BRIT, TAES, TEX, UC). King Co., 7 mi E of Guthrie along the South Fork of the Wichita Riv., Higgins 6255 (NCU, NY); South Llano River State Park, 0.1 mile S of park headquarters along gravel road leading into Buck WMA, near plot #3, Sanchez 1035 (BRIT). Kinny Co., Kickapoo State Park, Keeney 8708 (BRIT). Kleberg Co., Along Hwy 141 ca 3 mi W of Kingsville, Riherd 52–2 (TAES); 8.3 mi NE of Riviera, Parks & Cory 16968 (TAES); 1 mi N of Kingsville, Brothers 69 (TAES); King Ranch, Kingsville, Perdue 1623 (ISC, LL); 3.2 mi SE of Kingsville, Cory 16969 (GH, TAES). Live Oak Co., 7.6 mi S of George West, Johnston 542072 (BRIT, TEX). Llano Co., 17.4 mi W of Llano, Gould 8380 (TAES, UC); Enchanted Rock State Park, 19 mi N of Fredricksburg, Kast 78 (TAES); 4 mi S of Llano on Hwy, Heinemann 63–21 (TAES); Black Rock Park, Box 113 (TAES); Lower slopes of Enchanted Rock, Butterwick & Lamb 3360 (TEX); Just W of Enchanted Rock, Butterwick & Lamb 3089 (TEX); Ca 1 mi NE of Enchanted Rock, just W of Sandy Ck., Butterwick & Lamb 3296 (TEX); Enchanted Rock, Tharp s.n. (TEX). Lubbock Co., Lubbock, Texas Tech grass garden, Caddell s.n. (BRIT); Lubbock Lake Landmark State Historic Park, Launchbaugh s.n. (BRIT). Mason Co., Ca. 6 mi N of Mason on old Katemcy Rd., Waller 2156 (TAES); Katemcy, Ruegner s.n. (TAES); 13 mi W of Mason near road to Junction, Gould 5702 (TAES). Maverick Co., 8 mi N of Quemado, Gould 11319a (UC); 8 mi N of Quemado, Gould 11319b (TAES, UC, US). McMullen Co., Off Hwy 5.2 mi W of Tilden, Swallen 10008 (US); 9 mi W of Tildenon Rd. No. 63, Tharp & Johnston 541771 (TEX); 2.5 mi S of Tilden on Rd. no. 173, Tharp & Johnston 541779 (TEX). Nueces Co., Corpus Cristi, North Beach, Jones 1336 (FSU); 5 mi NW of Corpus Cristi, Parks & Cory 16970 (TAES); 2.5 mi S of Violet, Bockholt 52–22 (TAES); 0.4 mi W of junction roads 666 & 624 N of Banquete, Johnston 542329 (TEX); vacant lot on North Beach, Corpus Cristi, Jones 1336 (BRIT). Mitchell Co., Caliche Hill, NE 1/4 sec. 17, S.P.R.R. Block 18, Pohl 4622 (BRIT). Oldham Co., Adrian, waste area & ditches bordering Hwy on N side, Brooks & McGregor 16524 (GA, KANU, RM). Palo Pinto Co., Mineral Wells, Reed 34873 (TAES); Possum Kingdom State Park, near headquaters, Correll & Correll 24120 (LL, MO). Pecos Co., 6 mi W of Longfellow, Sanderson Canyon, Warnock 11848 (BRIT, LL); 29 mi W of Sheffield, Gould 7215 (TAES, TEX); Ca 6 mi N of Ft. Stockton, Warnock 46783 (BRIT, NA, TAES, TEX); “Roadside”, Tharp 43A71 (GH, MO, RM, UC, US). Presidio Co., Along US Hwy 67 11.2 mi S of Marfa, Spellenberg & Spellenberg 3695 (NY); 1/4 mi N of Solitario Peak, Johnston 3435 (TEX); Ridge just W of mouth of Musgrave Canyon, Tierra Viejas, Hinckley 1940 (GH, TEX); Ca. 1 mi W of mouth of San Antonio Canyon, Butterwick & Lott 3845 (LL); Along Cibolo creek near large Hwy bridge at Presidio, Warnock 96 (TEX, US); About 10.5 mi SE of Valentine on US Route 90, Van Devender s.n. (ARIZ 238886). Randall Co., Palo Duro Canyon, ca. 2 mi NE of Canyon off Interstate–87, Higgins 11374 (NY); S of Canyon, along creek bottom & breaks, Higgins 18579 (NY); Just E of Canyon near the water tower, Higgins 16974 (NY); Coyote Springs, Palo Duro State Park, Cory 50325 (BRIT). Reeves Co., Along US Hwy 290, 1 mi E of jct of US Hwy 80, Johnston 3285 (TEX); Balmorhea, Bayles 15420 (GH, TAES). Refugio Co., 8.5 mi SW of Woodsboro, Hill 7824 (TAES); Along Hwy 77, 0.7 mi N of the Aransas Riv. Crossing, Hill 7837 (TAES). San Patricio Co., Near entrance to Lake Corpus Cristi State Park, Gould 10971 (TAES); 7 mi S of Taft, Jones 1049 (BRIT); Lake Corpus Cristi State Park near Mathis, Brown 9525 (ARIZ). San Saba Co., 2 mi S of San Saba, Smith 24 (TAES); 20 mi SW of San Saba on Hwy 501, Hatch & Briggs 1206 (TAES); US Hwy 45, 4 mi N of Richland Springs, England & McCart 9135 (LL, TAES); Coleman Ranch, 3 airline miles SE of San Saba, Cory 58254 (BRIT). Schleicher Co., 11–3/4 mi N of Eldorado, Cory 52507 (BRIT, MICH). Scurry Co., 5 mi N of Fluvana, Correll & Johnston 17200 (LL, US); Caprock, ca. 4 mi N of Fluvana, Correll 15170 (BRIT, LL, MO, NY). Shackelford Co., Fort Griffin State Park, 9/10 mi W of south entrance, Cory 58428 (BRIT). Starr Co., 0.7 mi E of Rd. No. 755 on the side road to McCook (Hidalgo Co.), Tharp & Johnston 541895 (TEX). Sterling Co., Hill, Tharp s.n. (ARIZ 31152). Stonewall Co., 13 mi S of Aspermont & 3 mi S of Double Mt. Fork of Brazos Riv., on road to Hamlin, Johnston & Walker 6772 (LL). Sutton Co., Sonora branch of TAMU Experimental Station, Tunnell 53 (TAES); 13 mi W of Sonora, Gould 9691 (BRIT, TAES, UC). Tarrant Co., US Hwy 287 X Eden Rd., Snowden 915308 (TAES); Arlington, US Hwy 287 N–bound access road, between Eden & Sublett overpasses, Snowden 915218 (TAES). Taylor Co., Buffalo Gaps Estates ca 12 mi S of Abilene, Henderson 61–912 (FSU); Camp Barkeley, Tolstead 5812 (BRIT, TAES); 4 mi NE of Lawn, Mahler 4115 (TAES); Abilene State Park, ca. 20 mi S of Abilene, Henderson 63–988 (BRIT, TEX). Terrell Co., 9 mi N of Dryden, Gould 9709 (TAES, UC); 6 mi E of Sanderson, Warnock 6817 (BRIT, LL, TEX); Blackstone Ranch, rocky mesa slopes 15 mi S of Sheffield, Webster 172 (TEX); 26.5 km NW of Lantry, 37.0 km SE of Dryden on U.S. 90, Reinga 2004-740 et al. (ARIZ); 4 mi W of Sanderson, Shinners 17380 (BRIT). Throckmorton Co., W. Matthews’ Lambshead Ranch, ca. 20 km S and 15 km N of Throckmorton, Cornelius 1168 (BRIT); Ca. 2 km S and 17 km E of Throckmorton (Jimmy Mieng’s Elm Valley Ranch), Cornelius 1704 (BRIT). Tom Green Co., Hwy 2288, 5 mi from intersection with Hwy 87, Hoover 70 (TAES). Travis Co., NE of intersection of FM 2222 & Lakewood Drive, Carr 4001 (TAES); 0.1 mi W of Oasis Bluff Rd. from its junction with Bullock Hollow near the community of Marshall Ford, Jones & Jones 1860 (TAES); Brackenridge Field Station, Austin, Baird 3772 (KANU); Near Bull Ck. Lodge, along FM 2222, Mears 997 (TEX); ca. 1.5 airmiles due N of W end of Tom Miller (Lake Austin) Dam, Carr 6161 (BRIT); McDonald Ranch, Higdon 121 (TEX, US); Onion Ck., Moon 134 (TEX); Watkins Ranch, Tharp et al. 49033 (KANU, TEX); Colorado Riv. flood plain, Armer 5301 (TEX); Mt. Barker, Austin, Innes 171 (GH). Uvalde Co., Montell, Palmer 13016 (UC, MO). Val Verde Co., 5 mi E of Comstock on Hwyt 90, Zuberbueler 73 (TAES); 11.2 mi W of Comstock on US Route 90, Van Devender 84-653 et al. (ARIZ); 9-1/4 mi N of Del Rio, Parks & Cory 20831 (TAES); 3 mi N of Juno, Gould 9954 (TAES); Seminole Canyon State Historical Park, Labus 4 (TEX); Devil’s Riv., Tharp 43072 (TEX); Devil’s Riv. bridge on Hwy US 90 (W of Del Rio), Gould 9748 (TAES, TEX); 5 mi E of Juno, Warnock 15066 (TEX). Webb Co., N of Laredo, Silveus 7557 (ARIZ, BRIT). Williamson Co., 0.5 mi N of Round Rock, Gould 8420 (TAES, UC). Winkler Co., Along Concho Bluff, 20 mi NE of Kermit, Collins 667 (BRIT). Young Co. Graham, Reverchon 3451 (GH, MO, NY, US). Zapata Co., 15 mi N of San Ygnacio, Cory 35815 (TAES).

### Disakisperma eleusine

(Nees) P. M. Peterson & N. Snow, Annals Bot. 109: 1327. 2012.

http://species-id.net/wiki/Disakisperma_eleusine

Figure 3A−P

Diplachne eleusine Nees, Fl. Afr. Austr. 255. 1841. *Uralepis eleusine* (Nees) Steud., Syn. Pl. Glumac. 1: 248. 1854. *Tridodia eleusine* (Nees) T. Durand & Schinz, Consp. Fl. Afr. 5: 877. 1894. *Leptochloa eleusine* (Nees) T. A. Cope & N. Snow, Novon 8: 78. 1998.

#### Type.

SOUTH AFRICA, Katrivierspoort, JF Drège 3906 (lectotype: B!, designated by Snow (1998a); isolectotype: P!).

#### Description.

Perennials. Culms 50–130 cm tall, 2.5–4.0 mm wide at base, round, erect, rarely geniculate and rooting from lower nodes, arising from fibrous roots or sometimes from a knotted crown, branching or not; nodes glabrous; internodes 5–30 cm long, soft, hollow. Leaf sheaths longer or shorter than the internodes, round or slightly flattened, glabrous on the sides and margins; collar green or tan; ligules 0.8–1.4 mm long, membranous, often dark brown near base, apically truncate, erose or fimbriate; blades (3–) 15–30 cm long, 5–7 mm wide, cauline, linear, flat but becoming involute on edges, glabrous to minutely but densely scabrous above, nearly glabrous to moderately scabrous below, not disarticulating at base, midrib prominent. Panicles to 65 cm long, 1.5–3.0 cm wide, exserted at maturity; branches (1–) 3–9, (3–) 5.5–10.5 cm long, alternate along rachis, steeply ascending to erect, rigid, minutely scabrous, the axils glabrous or with a few short hairs. Spikelets 4.7–9.2 mm long, pedicels mostly less than 0.5 mm long, usually imbricate, 5–8 (–10)-flowered; callus glabrous; lower glumes 2.8–4.1 mm long, membranous, narrowly ovate, scabrous on midnerve, acute; upper glumes 2.9–5.3 mm long, membranous, ovate, scabrous on midnerve, acute; lemmas 3.2–4.2 mm long, 3– or sometimes 4–5 nerved (at least basally), membranous, ovate, smokey white, bronze, light green, lateral nerves distinctly raised, sericeous along lower half of lateral nerves, midnerves and often between nerves, the hair tips clavicorniculate, apex obtuse and sometimes bifid, awnless and often erose, hyaline; paleas subequal to lemma, elliptic to ovate, membranous, with short clavicorniculate or nonswollen hairs between lateral nerves, apex broadly acute. Anthers 0.9–1.0 mm long, brown or yellow. Lodicules 0.7−0.8 mm long. Caryopses 1.7–2.0 mm long.

**Leaf anatomy.** Ellis (1977) reported C_4_ NAD-ME anatomy for *Diplachne eleusine*, which we confirm here. Primary, secondary, and occasionally (e.g., *Snow & Burgoyne 7021*[MO]) tertiary bundles are evident. Midrib present but lacking associated lacunae. Primary bundles easily distinguished from secondary bundles; tertiaries separable from secondaries based on much smaller diameter and absence of associated sclerenchyma girders. Primary and secondary bundles in fresh material projecting somewhat adaxially but not abaxially. Outer bundle sheath cells of primary and secondary bundles interrupted adaxially and abaxially. Extension cells occurring adaxially above primary and secondary bundles but more so over primaries. Sclerenchyma girders present adaxially and abaxially above primaries and secondaries (more so adaxially) but lacking from tertiaries. Colorless cells lacking between adjacent bundles; chlorenchyma continuous between all bundles. Bulliform cells between primary and secondary bundles but adaxial (above) to tertiary bundles [Vouchers: *Snow & Burgoyne 6941* (MO), *7021* (MO); photographs of cross sections seen by the first author and housed at PRE: *Ellis 2057*, *2058*, *2059*, *2106*, *3546*, *3868*].

**Stem anatomy.** Outer (subepidermal) ring of sclerenchyma present. Broadly triangular sclerenchyma girders (broadest near epidermis) arising from outer ring and projecting inwardly to connect with outermost ring of vascular bundles. Assimilatory tissue present, partially ringed by Kranz-like cells, in distinct patches between adjacent vascular bundles and associated triangular girders. Inner ring of sclerenchyma present, continuous, and several layers thick. Inner ring of vascular bundles scattered in the remaining cortical tissue but these mostly close to but not touching inner sclerenchyma ring. Cortical vascular bundles surrounded by sclerenchyma at least around their xylem-bearing portion [Voucher: *Halse 29* (MO)].

**Chromosome number.** Not known.

**Figure 3.**
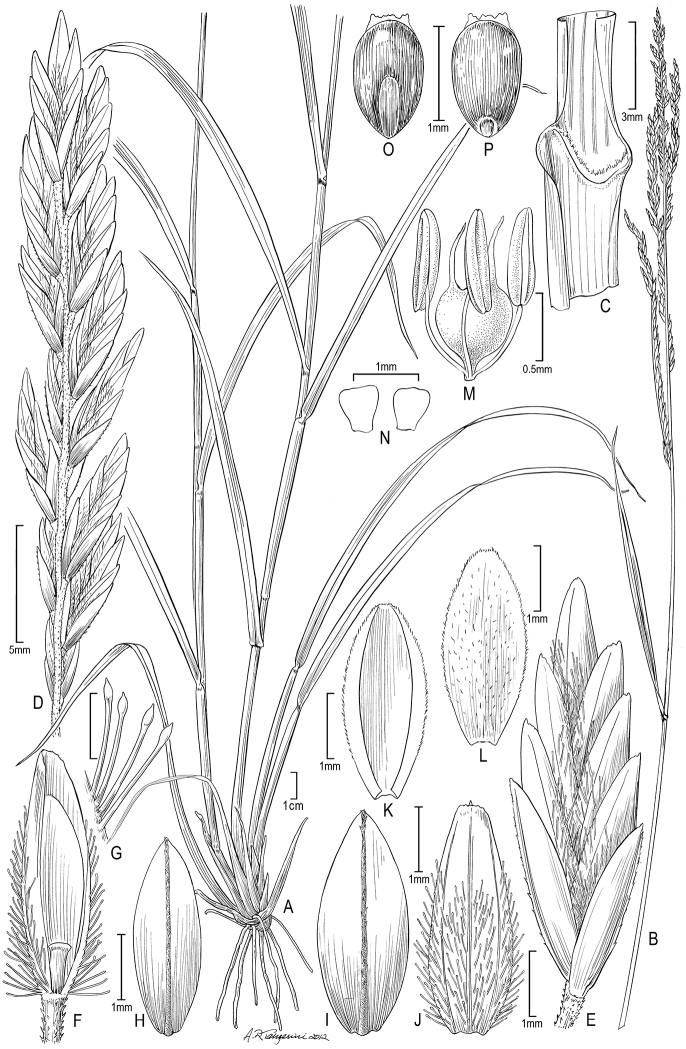
*Disakisperma eleusine* (Nees) P.M. Peterson & N. Snow **A** habit **B** culm andinflorescence**C** sheath, ligule, and blade, ventral view**D** branch of inflorescence **E** spikelet **F** floret, ventral view **G** clavicorniculate hairs **H** lower glume **I** upper glume **J** lemma, dorsal view **K** palea, ventral view **L** palea, dorsal view **M** stamens and pistil **N** lodicules **O** caryopsis, dorsal view **P** caryopsis, ventral view. Drawn from *Schweickert 1896* (US).

#### Phenology.

Flowering year-round.

#### Distribution.

**Native:** Africa south of about latitude 20^°^S and most common in South Africa; growing in heavy clays to sandy soils, in rocky open sites or disturbed woodland or bushveld, infrequent to common. Elevation ca. 300 to 2000 m. (TDWG: BOT, MOZ, NAM, CPP-WC, CPP-EC, CPP-WC, TVL-GA, TVL-MP, TVL-NP, NAT-OO, SWZ). **Non-native:** None known.

#### Comments.

*Disakisperma eleusine* resembles the partially sympatric *Disakisperma yemenicum*. Both species have relatively few and erect panicle branches that bear moderately to tightly overlapping spikelets, florets with distinctly raised lateral nerves, lemmas and upper glumes occasionally with basal remnants of extra nerves, clavicorniculate hairs and similar caryopsis morphologies (Phillips 1982; Snow 1996, 1998b). *Disakisperma yemenicum* is distinguished by having lemmas that are cartilaginous below with involute margins, leaf blades with scattered delicate, long hairs (3−5 mm long), smaller anthers 0.2−0.3 mm long, and shorter paleas (1/2–2/3 as long as the lemma). The base of the ligule is often dark brown in *Disakisperma eleusine*.

#### Conservation status.

Least Concern (IUCN 2010).

#### Etymology.

The epithet *eleusine* probably was used to suggest that the species resembles *Eleusine* Gaertn.

#### Vernacular name.

South African sprangletop (Snow 1997). Suggested name: Southern African Jacobsgrass.

#### Specimens examined.

Botswana. Central: Seleka Ranch, Hansen 3373(GABS). Mozambique. Lourenco Marques: Maputo, regiao de Changalane, próx. da Cabeca do Elefante, Myre & Carvalho 1847 (K). Maputo: Semi-riverine thicket, Hornby 2609 (K). Sul do Save: circ. Magude, entre Macaena e Panjano, Myre & Balsinhas 1572 (CANB, K); Prox. da povoacao da Moamba, Myre & Balsinhas 1628 (I); between Moamba & Boane, Schweichert 1896 (B, K, S, US); Entre Moamba & Ressano Garcia, Marques 2209 (K); Maputo, arredores de Catuanae, Myre & Carvalho 1434 (K). Namibia. Ausdauernde, aufrechte bis 1.30 m hohe Horste, Giess 8422, (M); ca. 25 mi E of Sesfontein, De Winter & Leistner 5884 (PRE). South Africa. Cape Districts, Sekoekoenieland, Stellenbosch, langs Olifansrivier, du Toit 124 (PRE); Dist. Kingwilliamstown, Kei Rd., Ranger 109 (PRE); Ft. Beaufort, Giffen 1630 (MO); Haha Haha Lagoon, Ellis 2106 (PRE); Fort Beaufort Distr., Killians’s grave, 5 mi NW of Fort Beaufort, Story 3875 (K); Dist. Queenstown, 38 mi SE by E of Queenstown, Acocks 17946 (K); Near Riv. Kei, Swallen 10575 (US); Albany [now merged with Cacadu], Lindsleath (sp.?) s.n. (US). East Cape District: Transkei, near the Kekau River, Drège 1840 (K). Kwazulu–Natal: Umzivulu, Schlechter 6424 (K); Ngotshe Distr., Farm Welcome, 42 km from Pongola on the road between Magudu & Candover, Koekemoer 125 (PRE); Louwsburg Distr., Itala Nature Reserve, Rangers house, Brown & Shapiro 166 (PRE); at False Bay, Godfrey & Bayer SH–1472 (PRE, US); Vryheid, Pole Evans 2641 (US); Slopes of Umkomaas Valley near Josephine Brodge, Otto 19993 (PRE); Ca 8.2 km S of Golela along hwy to Pongola, before “T” junction, Snow & Burgoyne 6954 (MO, PRE); Mkuze Game Reserve, A.J. Oates 1242 (US); Mkuze Game Reserve, Mahlobeni area, Snow et al. 6982 (MO, PRE); Ca 300 m from “T” intersection along dirt road leading to headquarters of Mkuzi Game Reserve, from dirt road leading S from town of Mkuzi, Snow & Burgoyne 6963 (MO, PRE); Zululand, Umfolozi Game Reserve, Guy & Ward 7 (PRE); Zululand, Prinshof Experimental Station, Pretoria, Phillips & Goossens 8785 (MEL, PRE, US); Zululand, Hluhluwe Game Reserve, Huntley 1735 (MO); Umtambanana, Halse 29 (MO, U); Nkansini, Tugela Valley, Smook 1817 (K, MO); N Weenen on Muden Rd, Acocks 13458 (BM, K); Inguavuma Dist., Ndumu Game Reserve, Pooley 1040 (K). Limpopo: Kruger N.P., Schÿff 3968 (K); Lebowa, Blouberg Mt., Farm Buffelshoek 261 on the SW side of the massif on the Blouberg geological series, Smook 7371 (PRE); Kruger N.P. 13 mi E of Skukuza, de Winter & Codd 529 (BM, K); Kruger N.P. 8 mi E of Skukuza, de Winter & Codd 524 (BM); Kruger N.P., 10 km east of Satara on Satara–Nwanedzi Rd. near Msasane Windmill, Ellis 3546 (PRE); Blaauwberg Tin Mine, Schweickerdt 2011 (B, BM, US); Lebowa: Stellenbosch, 56 km van Burgersfort op Pietersburg pad, draii NO af vir 10 km, du Toit 1001 (PRE); Noordelike kant van Soutpansberg, Nel 320 (PRE). Mpumalanga: Loskopdam Nature Reserve, along dirt road leading west from administration buildings, ca 2 km along road, Snow et al. 6941 (MO, PRE); Loskop Dam Nature Reserve, Ellis 2058 (PRE); Blyde Riv. Canyon Nature Reserve, north end of nature reserve, just off of Hwy R532, Snow et al. 7021 (MO, PRE); Bellevue, SO van Steilloopbrug, du Toit 161 (PRE); Distr. Potgietersrust, Springbuck Flats, de Winter 2331 (PRE). Swaziland. Farm Mlawula, below western edge Lubombo Mts, Upper Nkumbane Valley, small dam near Cyrildene fence, Culverwell 298 (PRE); 2–3 mi W of Border (with Natal) at Cecil Mack Pass, Maguire 8398 (M); Mlawula Nature Reserve, SARA campsite, Braun 119 (PRE).

### Disakisperma obtusiflorum

(Hochst.) P. M. Peterson & N. Snow, Annals Bot. 109: 1327. 2012.

http://species-id.net/wiki/Disakisperma_obtusiflorum

Figure 4A−O

Leptochloa obtusiflora Hochst. Flora 38: 203. 1855. *Eleusine obtusiflora* (Hochst.) Blatt., Rec. Bot. Surv. India 8: 505. 1936.

#### Type.

ETHIOPIA, W. Schimper in Herb. Buchinger 1204 (holotype: STR; isotypes: B!, P!, S! co-mounted with a specimen of *Trigonochloa uniflora* (Hochst. ex A. Rich) P.M. Peterson & N. Snow).

#### Description.

Perennials. Culms 45–200 cm tall, 1.2–5.0 mm wide at base, round, erect to sprawling, from fibrous or knotted root crowns sometimes bearing short cataphylls (rhizomatous in the opinion of some, e.g. *Chiovenda 2585* [US]), often branching (sometimes abundantly so; e.g., *Glover & Gilliland 298* [US]); nodes glabrous; internodes (1.5–) 3–35 cm long, soft, hollow. Leaf sheaths longer or shorter than the internodes, mostly round, glabrous or sparsely hairy near apex, the margins glabrous; collar tan or maroon; ligules 1.7–2.2 mm long, membranous, often somewhat lacerate, apically truncate and erose; blades 7–37 cm long, up to 12.5 mm wide, cauline, linear, flat but drying involute, mostly glabrous but sometimes sparsely pilose at the base of blade to minutely but densely scabrous above, nearly glabrous to minutely but densely scabrous below, not disarticulating at base, midrib prominent. Panicles 28–65 cm long, 3–8 (–12) cm wide, exserted at maturity; branches (4–) 9–18, (2.5–) 9–13 cm long, alternate along the rachis, steeply ascending to erect, somewhat flexuous, minutely scabrous, the axils shortly pilose. Spikelets 3.7–6.5 (–8.5) mm long with 5−10 (−15)-flowered, pedicels to 1.2 mm long, imbricate about halfway near base of branches to tightly at branch tips; callus glabrous; lower glumes 1.5–2.9 mm long, membranous, narrowly ovate or occasionally ovate, scabrous on midnerve, acute; upper glumes 1.8–2.9 mm long, membranous, ovate, scabrous on midnerve, acute; lemmas 2.2–2.8 (–3.0) mm long, 3-nerved (rarely 4–5-nerved above base), membranous, ovate, light brown, green, or maroon, the lateral nerves distinctly raised and usually bright green, sericeous along the lower ¾ of the nerves and between the nerves, the hair tips clavate or clavicorniculate, apex obtuse and sometimes bifid; paleas 2.0–2.3 mm long, membranous, ovate, sparsely short pubsecent on lower half between nerves or nearly glabrous, apex broadly acute. Anthers ca 0.7 mm long, yellow. Lodicules 0.2−0.3 mm long. Caryopses 1.4–1.5 mm long, 0.7–0.8 mm wide.

**Leaf anatomy.** Midribs are always present and the diameter of primary bundles exceeds that of the secondaries.

**Stem anatomy.** Culms with outer (subepidermal) and inner (cortical) sclerenchymatous rings. Other details uncertain [Voucher: *Harris & Tadres 2151* (MO)].

**Chromosome number.** *n* = 20 (Ammal 1955; voucher not seen).

**Figure 4.**
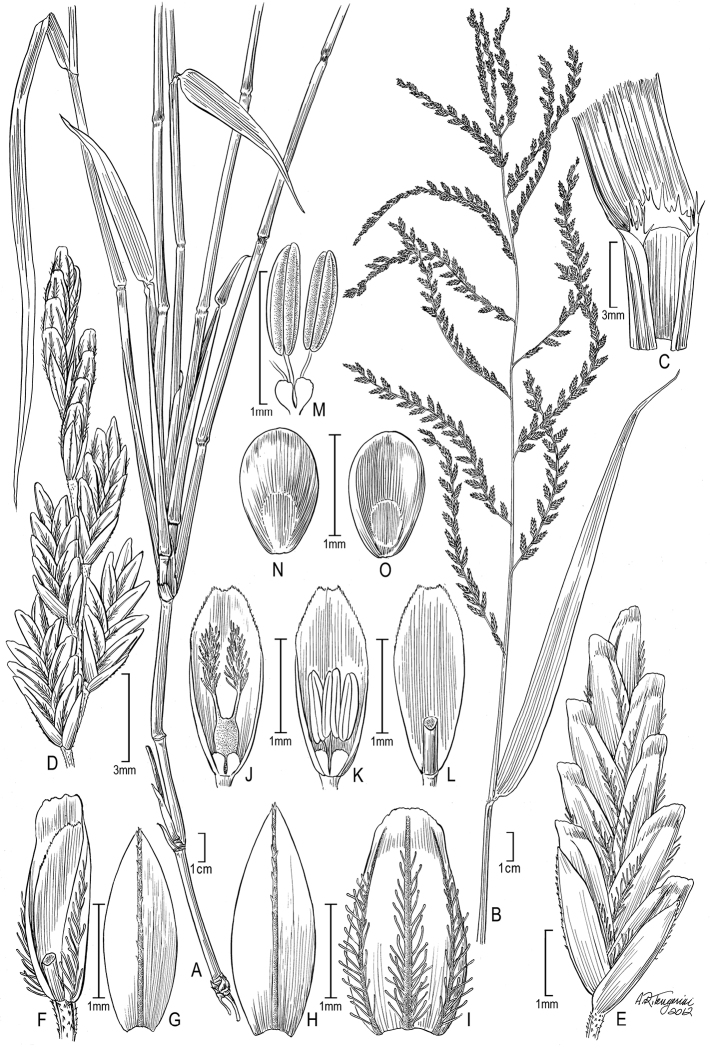
*Disakisperma obtusiflorum* (Hochst.) P. M. Peterson & N. Snow **A** habit **B** culm andinflorescence**C** sheath, ligule, and blade, ventral view**D** branch of inflorescence **E** spikelet **F** floret, ventral view **G** lower glume **H** upper glume **I** palea, dorsal view I lemma **J** lodicules and pistil enclosed in palea **K** lodicules and stamens enclosed in palea **L** palea, dorsal view **M** lodicules and stamens **N** caryopsis, dorsal view **O** caryopsis, ventral view. Drawn from *Shantz 791* (US).

#### Phenology.

Flowering July through December.

#### Distribution.

**Native:** India west to Saudia Arabia, Yemen and Somalia; south through Tanzania, and Angola; in a variety of semi–open or disturbed habitats in rocky areas or sandy to heavy soils, from sea level to 1900 m. (TDWG: ANG, BUR, CON, ERI, ETH, KEN, SAU, SOM, SUD, TAN, UGA, YEM. **Non-native:** IND (see comment below).

#### Conservation status.

Least Concern (IUCN 2010).

#### Etymology.

The epithet probably refers to the obtuse lemma apex.

#### Vernacular name.

North African sprangletop. Somalia: Buldorle, Rareh. Suggested: African Jacobsgrass.

#### Comments.

Herbarium specimens (and label descriptions) often indicate the panicle as nodding or pendulous, which contrasts with the more nearly erect panicle of the related *Disakisperma eleusine*, which *Disakisperma obtusiflorum* closely resembles, but which occurs farther. The collection of *Jackson 3977* (K)differs in its short stature, profuse branching, and short panicles that bear only 1–2 branches, which may reflect local grazing selection. Over ninety collections have been seen from Uganda, although not all are reported below.

The species evidently may occur in nearly pure stands and is known in abandoned cultivated areas. The label of *Greenway 2213* (US) indicates the species is “A useless fodder as it causes dysentery in cattle according to the Wapares.” Boonman (1993) reports that *Disakisperma obtusiflorum* is a potential species for reseeding denuded pasture.

Hooker (1896) clearly included *Poa maysorensis* Rottler ex Hook. f., nom. inval., in synonymy of *Disakisperma obtusiflorum*, andmerely cited the Rottler name as being in manuscript. Hooker (1896) suggested that the presence of the species in southern India in Kochi (“Cochin”) was an introduction due to heavy trade between that port and Africa.

#### Specimens examined.

Angola. Loanda: Maianga d’El Rei, Welwitsch 7282 (BM); On the rocky banks of Riv. Long at Capolo, Gossweiler 8281 (K, US). Burundi. Bubanza: Rugombo, Reekmans 6486 (B, MO, PRE). Bujumbura: Gitaza, Reekmans 4010 (MO). Democratic Republic of the Congo. Sud-Kivu. Kabare, Bequaert 5348 (K); Parq. Nat. Albert [=Virunga Nat. Park], Rurindi, Lebrun 7943 (K, M, MO) & Rurindi, Lebrun 7784 (MO, NY). Orientale. Terr. Mahagi, Plaine Iswa–village Awora, Taton 1246 (MO). Eritrea. Northen Red Sea: Lungo il Torrente Haddas, Pappi 2585 (G, MO, P, US). Ethiopia. Harrar: 35 km along the road from Dire Dawa to Erer Gota, De Wilde 6449 (M, MO). Gamu Gofa: 72 km S of Soddu on road to Arba Minch, 50 km from Arba Minch, Gilbert et al. 8860 (K). India. Kerala: Cochin (=Kochi), Rottler s.n. (K). Tamil Nadu: Coimbatore, Narayana 1929 (NY); On the way to Areakhalti, Raju & Napanatham 4763 (K). Madras [Chennai], Janaki Ammal 1154a (K); Masanagudi, The Niligris, Barber 2656 (US). Kenya. Coast Province, Kwale, near Taru, between Samburu & Mackinnon Rd., Drummond & Hemsley 4192 (B); Tsavo N.P. West, 7 km S of Mtito Andei, Belsky 527 (BH); Kitovo, Taveta, Nye 42 (BM); Mtwapa, Thorold 2002 (K); Vipingo 20 mi N of Mombasa, Verdcourt 1101 (K, MO). Mombasa, Napier 6370 (K, PRE); Tsavo N.P. East, Greenway & Kanuri 12836 (K, P); Tsavo N.P., N end, Heady 1759 (UC); Gazi, along coast, Heady 1376 (MO, UC); Galole [=Hola], Makin 128 (P). Eastern Province, Kiboko Range Research Stn., near Makindu, Ndegwa 579 (MO); Near Makindu Riv.,... Kiboko Range Research Stn., Ndegwa 474 (MO); Kibwezi, McCallum Webster K164 (K); Kilifi Distr., N of & just below Jilore Forest Station, Perdue & Kibuwa 10124 (K, NA); Maktau, Hitchcock 24697 (K, US). Nairobi Province, Nairobi, Hitchcock 24822 (US); Nairobi, Lyne Watt 18 (US). Nyanza Province, Kisumu, Hitchcock 24865 (K, US). Rift Valley Province, K1, Mathew’s Range [=Lenkiyio Hills], Gachathi 440 (B); Southern Turkana at Namorutung’a near Lokori, Mwangangi & Gwynne 1161 (MO); Kitale grass nursery, Heady 1474 (MO, UC); Turkana Distr., Oropoi Valley, Liebenberg 27 (MICH, PRE, US). Saudi Arabia. Wadi Maraba, foot of Raidah Escarpment, Collenette 8764 (K); Jizan–Abha Rd., ca. 20 km from Abha, Chandhanj 7262 (K). Somalia. Government House grounds, Hargeisa, Glover & Gilliland 17 (BM, K); Within a radius of ca. 5 mi of the Hargeisa Stn, Farquharson 29 (K); 29 km SW on coast road from Mogadishu airport, then inland 2.5 km, Kuchar 17708 (K); Burran, Erigavo Distr., McKinnon S122 (BM, US); Between Wasdere & Walwal, Glover & Gilliland 398 (BM, P, US); Wobleh, Gillett 4584 (K, P); Tugdheer region, 6 km NW of Borao, Hansen & Heemstra 6105 (K). Hargesia, Gillett 4059 (K, P, S). Sudan. Kurdufan Province, Rahad, 1 hour S of J. Hadadiad, Taganor Wilderness, Michelmore s.n. (K). Red Sea Province, Slopes Jebel Hamoyet, Red Sea Hills, Jackson 3977 (K). Tanzania. Arusha Region, Lake Manyara N.P., Near Mbagaya Riv.-Ndabash, Greenway & Kanuri 11280 (K, PRE); Serengeti N.P., Serengeti Research Institute, Belsky 144 (BH, MO). Kilamanjaro Region, Moshi, GB Wallace 1190 (NY); Mwanga District, Peterson, Soreng & Romaschenko 24198 (DSM, US). Lindi Region, Lutamba–See, 40 km westlich Lindi, Schlieben 5945 (M, S); Lindi, H J Schlieben 5945 (M). Manyara Region, Mbagaya Riv.-Ndabash, Lake Manyara N.P., Greenway & Kanuri 11280 (PRE). Mara Region, T1, Klein’s Camp to Bologonja Riv., Greenway 10666 (K); Musoma Distr., Seronera, Greenway 9971 (K, US). Moshi Region, Pangani Distr., Kikokwe area, Bond 84 (P); Pangani Distr., Jassini in sandy hollow, Milne–Redhead & Taylor 7297 (K, US); Moshi, Hitchcock 24557 (US); Vicinity of Moshi, Kiboscho Rd., Piemeisel & Kephart 405 (US); Moshi Town, B Mhoro 1868 (MO); Moshi Kilimanjaro, Shantz 791 (K, US). Mwanza Region, Lake Prov., Nyegezi, Bunegeji Chiefdom, Tanner 1060 (K, MICH, NY, UC). Tanga/Kilimajaro Region, Pandani River, Peterson, Soreng & Romachenko 24243 (DSM, US). Tanga Region, Korogwe Distr., Magunga Estate, Faulkner 961 (A, K); 5 km W of Tonibombo, Peterson, Soreng & Romaschenko 24211 (DSM, US); Muheza, Hitchcock 24540 (US); Mkaramo, Mkwaja Subchiefdom, Pangani Distr., Tanner 2346 (K, NY, UC). Zanzibar Central/South Province, Kisuani [Airport], Greenway 2213 (K, US). Unknown Province: T3, Kwamarukanga, Magogo 344 (K); Saira, HG Faulkner 3775 (K, P). Uganda. Central Region, Mengo–Kisenyi, Kyadondo, W. Mengo District, Rwaburindore 2134 (MO). Eastern Region, Buyonjo, Lake side, Busogo, Maitland 1100 (US); Serere, at Robori, Maitland 1345 (B, K, US). Western Region, Katwe Ruwenzori, Maitland 963 (B); Butiala, Lake Albert, Thomas 4086 (K); Mohokya Toro, AS Thomas 2757 (K). Yemen. Between Beni Omer & Barakain, Hoogariah, Wood 1407 (BM, K); Between Taiz & Sharab (Roona), Wood Y/75/966 (BM); Hadia, Wood Y/75/797 (BM).

### Disakisperma yemenicum

(Schweinf.) P.M. Peterson & N. Snow comb. nov.

urn:lsid:ipni.org:names:77131997-1

http://species-id.net/wiki/Disakisperma_yemenicum

Figure 5A−Q

Eragrostis yemenica Schweinf., Bull. Herb. Boissier 2 (App. 2): 41. 1894. *Cypholepis yemenica* (Schweinf.) Chiov., Annuario Reale Ist. Bot. Roma 8(3): 357–358. 1908. *Eleusine yemensis* (Schweinf.) Chiov., Ann. Bot. (Rome) 10: 410. 1912. *Coelachyrum yemenicum* (Schweinf.) S.M. Phillips, Kew Bull. 37(1): 159. 1982.
Leptochloa appletonii Stapf, Bull. Misc. Inform. Kew 6: 223. 1907. TYPE: SOMALIA. Golis Range, Drake-Brockman 147 (lectotype: G (photo)!, designated here).
Eragrostis diplostachya Peter, Repert. Spec. Nov. Regni Veg. Beih. 40 (1, Anhang): 100, t. 58, f. 1. 1929. TYPE: TANZANIA. Lushoto District, Buiko, Peter 11083 (lectotype: B designated by S.M. Phillips, 59. *Cypholepis*, Gramineae, part 2, Fl. Trop. E. Africa 250. 1974; K-photo!).

#### Type.

YEMEN.Arabia Felici, 2 Feb 1889, G.A. Schweinfurth 1332 (lectotype: G-photo! designated here, S.A. Chaudhary, Grasses of Saudi Arabia 274. 1989, earlier cited no specific herbarium). We are choosing the G specimen as the lectotype because it is easily seen (electronically), was previously selected by Chaudhary (1989), and the orginal collection at B was destroyed.

#### Description.

Perennials. Culms 30–100 cm tall, 1–2 mm wide at base, usually flattened, erect, infrequently decumbent or sprawling, arising from fibrous roots, culms unbranched or only as tillers from very base; nodes glabrous; internodes 4–14 cm long, soft, hollow. Leaf sheaths longer or shorter than the internodes, round or slightly flattened, glabrous on the sides and margins; collar green or tan; ligules 0.5–1 mm long, membranous, erose or lacerate, apex truncate; blades 7–32 cm long, 2.5–5.5 mm wide, cauline, linear, flat but becoming folded to loosely involute, scaberulous with scattered, delicate, straight hairs near base above, the hairs 3−5 mm long, glabrous to scaberulous below, often disarticulating at base, midrib prominent. Panicles 3.5−19 cm long, 1.0–3.0 cm wide, exserted at maturity, composed of 2−8 racemosely arranged branches; branches 2–7 cm long, alternate along rachis, rachis often somewhat zig-zaged from each spikelet insertion to the next, steeply ascending to erect, rigid, axis scabrous. Spikelets 5–10 mm long, pedicels mostly less than 0.5 mm long, usually imbricate, 7–12-flowered; callus glabrous; glumes 2−3.8 mm long, subequal, 1-nerved, membranous, lanceolate to narrowly ovate, scabrous on midnerve, the midnerve dark green; apex acute to obtuse; rachilla joint cartilaginous and expanded above; lemmas 2.5–4.7 mm long, usually 3-nerved, membranous above, lower margins cartilaginous and involute, ovate, pale green with dark green nerves, often tinged with dark or olive-green, glabrous above and appressed pilose on lower half, the hairs clavicorniculate, apex obtuse to subacute, awnless; paleas 1/2−2/3 as long as the lemma, ovate, longitudinally bowed-out, the keels ciliolate, dorsal surface glabrous or with appressed clavicorniculate hairs on the lower half, apex obtuse. Anthers 0.2–0.3 mm long, yellowish. Lodicules about 0.2 mm long. Caryopses 1.2–1.5 mm long, 0.7−0.9 mm wide.

**Leaf anatomy.** Watson and Dallwitz (1992) reported C_4,_ XyMS+ anatomy with centripetal chloroplasts. Midribs conspicuous with colorless cells, having a conventional arc of bundles (a large bundle, flanked on each side by two smaller bundles). All vascular bundles accompanied by sclerenchyma (Watson and Dallwitz 1992; Snow unpublished). Bulliforms present in simple fans.

**Stem anatomy.** Not known.

**Chromosome number.** Not known.

**Figure 5.**
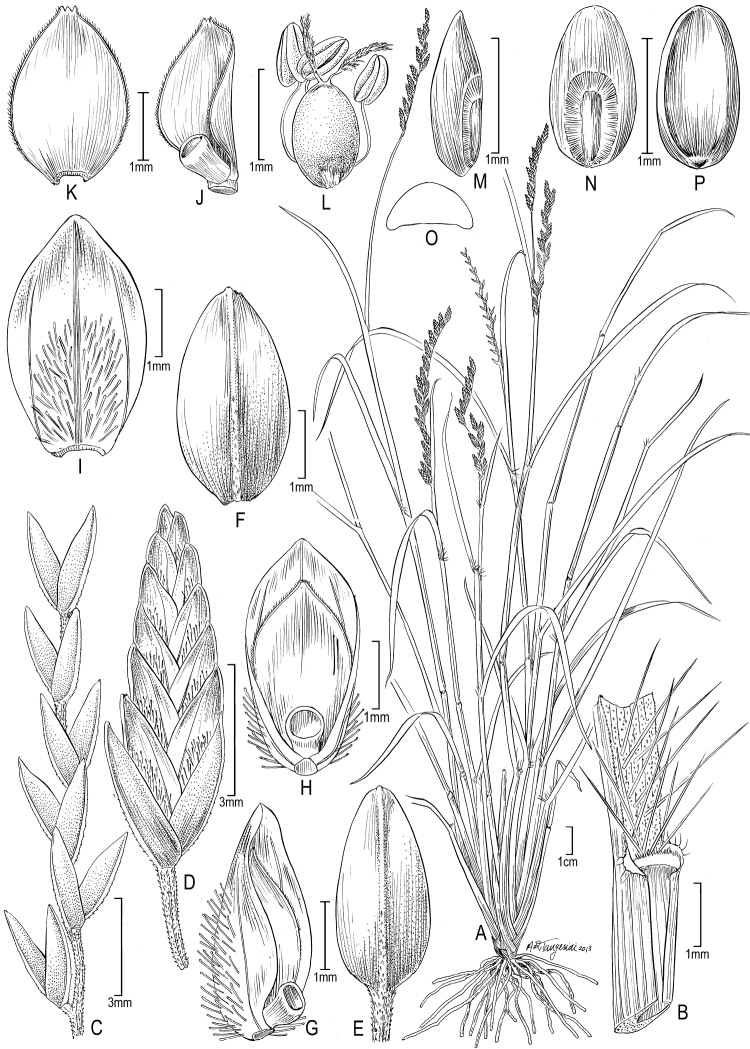
*Disakisperma yemenicum* (Schweinf.) P.M. Peterson & N. Snow **A** habit **B** sheath, ligule, and blade, ventral view**C** branch of inflorescence with glumes **D** spikelet **E** lower glume **F** upper glume floret, ventral view **G** floret, lateral view **H** floret, central view**I** lemma **J** palea with rachilla joint **K** palea, ventral view **L** pistil and stamens **M** caryopsis, lateral view **N** caryopsis, dorsal view **O** caryopsis, cross section **P** caryopsis, ventral view. Drawn from *Peterson*, *Soreng & Romaschenko 24254* (US).

#### Phenology.

Flowering July through December.

#### Distribution.

**Native:** Saudia Arabia, Oman, Yemen, Eritrea, Ethiopia, Somalia, Kenya, Tanzania, and South Africa; growing in *Acacia* and miombo woodlands on dry, shallow, often stoney soils among rocks, often in disturbed habitats (Phillips 1974b). Elevation 250−2100 m. (TDWG: CPP, ERI, ETH, KEN, OM, SAU, SOM, TAN, YEM.)

**Non-native:** Not known.

#### Conservation status.

Least Concern (IUCN 2010).

#### Etymology.

The epithet refers to Yemen, the geographical origin of the holotype.

#### Vernacular name.

Suggested: Yemen’s Jacobsgrass.

#### Comments.

*Disakispermum yemenicum* most closely resembles *Disakisperma eleusine* by virtue of the ascending to erect panicle branches, but the base of the lemma of *Disakisperma yemenicum* is cartilaginous, the base of the leaf blade adaxially has delicate straight hairs mostly 3−5 mm long, and its anthers are less than 0.5 mm long (vs. 0.9−1.0 mm in *Disakisperma eleusine*). We have examined relatively few specimens of *Disakisperma yemenicum* given that initital herbarium studies did not believe it was a part of *Leptochloa* s.l. (Snow 1997).

#### Specimens examined.

Eritrea.Assaorta, A. Pappi s.n. (US); Ocule, A. Pappi 5239 (US). Kenya. 6 mi SW of Nairobi, Nairobi National Park, S.L. Hatch 4220 (TAES, US). Somalia. Buramo, J.B. Gillett 4872 (US); Daganyado, P.E Glover & H. Gilliland 745 (US). South Africa. Cape Province, Boetsap Barkly West, A. Brueckner 143 (US); Kimberley Div. M. Wilman s.n. (US); Farm Rosenthal Mopane, L.E.W. Codd 4455 (US); North Cape, A. Brueckner 1032 (US). Tanzania. Shinyanga Region, Serengeti National Park, Naabi Hill, Peterson, Soreng & Romaschenko 24254 (DSM, US).

##### Excluded names

*Leptochloa digitatiformis* Beetle, Phytologia 52: 14. 1982. TYPE: Mexico, Sonora, Mpio. Fronteras, ejido km 47 km, Bernal & Cuadra s.n. (holotype: SARH, A. Beetle pers. comm. to N. Snow in 1993, but not found there by Snow).

#### Comment.

The status of this taxon is not entirely certain since we cannot locate the type for study (see comments above under species).

*Diplachne dubia* var. *humboldtiana* Kuntze, Revis. Gen. Pl. 3(2): 349. 1898. *Leptochloa dubia* var. *humboldtiana* (Kuntze) Beetle, Phytologia 54: 4. 1983. TYPE: Mexico.

#### Comment.

We have been unable to locate a type for this name (but not at NY; P.M. Peterson, pers. obs., 2013).

## Supplementary Material

XML Treatment for Disakisperma

XML Treatment for Disakisperma dubium

XML Treatment for Disakisperma eleusine

XML Treatment for Disakisperma obtusiflorum

XML Treatment for Disakisperma yemenicum
